# Small Extracellular Vesicles Have GST Activity and Ameliorate Senescence-Related Tissue Damage

**DOI:** 10.1016/j.cmet.2020.06.004

**Published:** 2020-07-07

**Authors:** Juan Antonio Fafián-Labora, Jose Antonio Rodríguez-Navarro, Ana O’Loghlen

**Affiliations:** 1 Epigenetics & Cellular Senescence Group, Blizard Institute, Barts and The London School of Medicine and Dentistry, Queen Mary University of London, 4 Newark Street, London E1 2AT, UK; 2 Instituto Ramón y Cajal de Investigaciones Sanitarias, Neurobiología-Investigación, Hospital Ramón y Cajal, Ctra Colmenar km 9.1, 28034 Madrid, Spain

**Keywords:** extracellular vesicles, EV, senescence, aging, senescence-associated secretory phenotype, SASP, glutathione-S-transferase, GST, lipid peroxidation, rejuvenation, ROS, 4-HNE, glutathione metabolism, GSH

## Abstract

Aging is a process of cellular and tissue dysfunction characterized by different hallmarks, including cellular senescence. However, there is proof that certain features of aging and senescence can be ameliorated. Here, we provide evidence that small extracellular vesicles (sEVs) isolated from primary fibroblasts of young human donors ameliorate certain biomarkers of senescence in cells derived from old and Hutchinson-Gilford progeria syndrome donors. Importantly, sEVs from young cells ameliorate senescence in a variety of tissues in old mice. Mechanistically, we identified sEVs to have intrinsic glutathione-S-transferase activity partially due to the high levels of expression of the glutathione-related protein (GSTM2). Transfection of recombinant GSTM2 into sEVs derived from old fibroblasts restores their antioxidant capacity. sEVs increase the levels of reduced glutathione and decrease oxidative stress and lipid peroxidation both *in vivo* and *in vitro*. Altogether, our data provide an indication of the potential of sEVs as regenerative therapy in aging.

## Highlights

• sEV-Ys ameliorate certain cellular biomarkers of senescence and aging
• GSTM2 is enriched in sEV-Ys in comparison with sEV-Os
• sEV-Ys have intrinsic GST activity increasing GSH levels in mice and humans
• sEV-Ys decrease ROS accumulation and lipid oxidation in old mice and human fibroblasts

## Graphical Abstract

**Figure:**
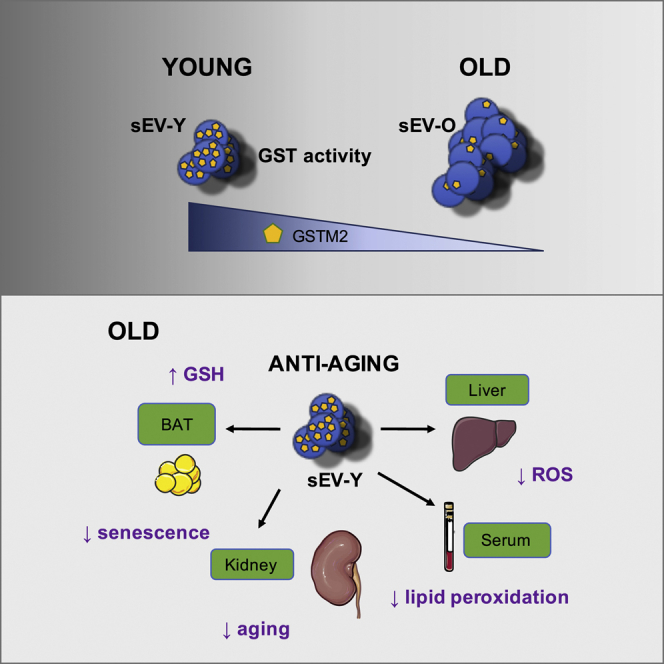

## Teaser

Fafián-Labora et al. demonstrate that small extracellular vesicles isolated from young human donor fibroblasts (sEV-Ys) possess glutathione-S-transferase (GST)-intrinsic activity. Through this activity, sEV-Ys ameliorate senescence and aging by increasing the antioxidant capacity of old fibroblasts and old mice, thus preventing lipid peroxidation.

## Context and Significance

**In this work, we investigate novel strategies that could lead to improving wellbeing throughout life. As blood from young mice has the ability to rejuvenate old mice, we wanted to determine if extracellular vesicles (EVs), balloon-like structures released by cells, were involved. We find that EVs from young donor cells rejuvenate certain features of aging in mice and human cells, as they are very effective natural antioxidants. At present, there is a huge effort to determine pharmacological strategies to achieve rejuvenation, but most drugs are toxic. Instead, EVs are a natural source and do not induce toxicity or immunogenicity; thus, this study opens new avenues for strategies to improve lifelong health and wellbeing.**

## Introduction

Aging is described as the functional time-dependent decline in tissue homeostasis affecting several organs in an individual. This decline is mediated by the activation of certain molecular and cellular signatures that comprise two of the main hallmarks of aging: disrupted intercellular communication and activation of the cellular phenotype termed senescence (López-Otín et al., 2013). Although no single biomarker for the identification of senescent cells has been identified so far, the main characteristics are a stable exit from the cell cycle and a compromised intercellular communication termed senescence-associated secretory phenotype, or SASP (Coppé et al., 2010, Fafian-Labora and O’Loghlen, 2020, Kuilman et al., 2010, Lee and Schmitt, 2019, Muñoz-Espín and Serrano, 2014).

The SASP is mainly formed by soluble factors, matrix remodeling enzymes, and extracellular vesicles (EVs) (Borghesan et al., 2019, Coppé et al., 2010, Fafian-Labora and O’Loghlen, 2020, Jeon et al., 2019, Takahashi et al., 2017). It is hypothesized that chronic SASP is the main culprit for inducing aging and age-related disease. Although the mechanisms involved are not well characterized, it is thought to be partially due to non-cell-autonomous signaling (Acosta et al., 2013, Borghesan et al., 2019, McHugh and Gil, 2018).

Senescence can also favor partial tissue reprogramming *in vitro* and *in vivo*, which in turn ameliorates cellular and physiological hallmarks of aging through epigenetic remodeling and soluble factors (Liu et al., 2011, Mosteiro et al., 2016, Ocampo et al., 2016). Interestingly, this complements previous studies where blood-borne factors were shown to rejuvenate mice of old age (Conboy et al., 2005, Scudellari, 2015, Villeda et al., 2014). Altogether, these findings show the potential of non-cell-autonomous signaling in ameliorating age-associated hallmarks.

It has been recently shown that small EVs (sEVs) play a previously uncharacterized role in senescence, aging, and age-related diseases (Basisty et al., 2020, Borghesan et al., 2019, Jeon et al., 2019, Takahashi et al., 2017, Yoshida et al., 2019); thus, here we aim to determine whether sEVs could also be implicated in ameliorating biomarkers of senescence and aging *in vitro* and *in vivo*. Our data show that sEVs derived from fibroblasts isolated from young human healthy donors (sEV-Ys) have the ability to prevent biomarkers of senescence characteristic of old human donors, cells undergoing oncogene-induced senescence, and fibroblasts derived from Hutchinson-Gilford progeria syndrome (HGPS), a severe accelerated form of aging. Interestingly, although the soluble fraction (SF) also ameliorated the proliferation arrest characteristic of senescence, its effect was modest in comparison with sEVs while large EVs (lEVs) displayed no functionality. Importantly, we show that sEV-Ys have intrinsic GST activity in contrast with the SF and have increased protein expression levels of glutathione-S-transferase mu 2 (GSTM2). Furthermore, sEV-Ys have the ability to reverse the accumulation of reactive oxygen species (ROS) and prevent lipid peroxidation *in vitro* and in certain organs in mice of old age. Altogether, we show that sEV-Ys can ameliorate a variety of features of senescence and aging *in vitro* and *in vivo* in certain organs.

## Results

### Small Extracellular Vesicles Isolated from Young Human Donor Fibroblasts Ameliorate Biomarkers of Senescence *In Vitro*

**Figure 1:**
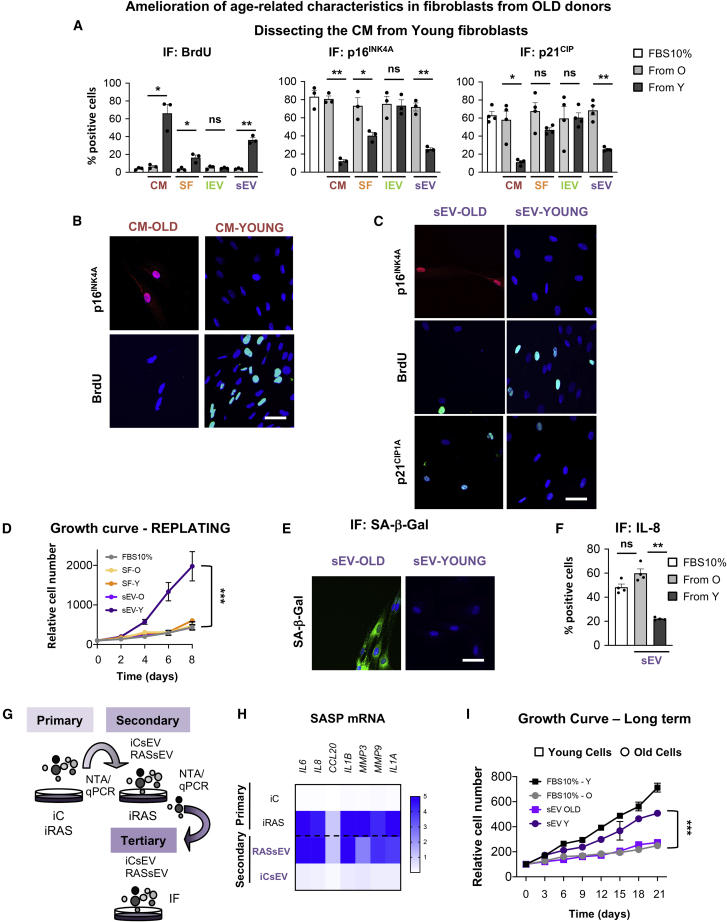
Functional Analysis of Different Fractions of the Conditioned Media of Young Donor Cells on Old Recipient Fibroblasts (A) Three (AG06240, AG13152, and AG13222) to four (AG16086, AG06240, AG13152, and AG13222) different cells derived from old donors were treated with pooled conditioned media (CM) from 3–4 young cells and separated fractions (SF, soluble fraction; large and small extracellular vesicles, lEVs and sEVs, respectively). Quantification of the percentage of cells staining positive for markers of senescence: proliferation measured by BrdU incorporation (left), p16^INK4A^ (middle), and p21^CIP1^ (right) staining. Data represent the mean ± SEM of 2 independent experiments in 3–4 old cell lines. FBS 10% represents cells treated with medium as control. t test analyses were performed. ∗p < 0.05; ∗∗p < 0.01; ns, non-significant. (B) Representative pictures of p16^INK4A^ and BrdU staining by IF in the old cell line AG16086 treated with CM from old (CM-OLD) or young cells (CM-YOUNG). (C) Representative pictures of AG16086 (old fibroblast) treated with sEVs derived from old (sEV-OLD) or young (sEV-YOUNG) cells. (D) Growth curve representing the mean ± SEM of the relative cell number of 4 old donor cell lines treated with either SF or sEVs from young or old donors at different time points shown in days. FBS 10% is control. Two-way ANOVA was performed. ∗∗∗p < 0.001. (E and F) Representative pictures of senescence-associated β-galactosidase (SA-β-Gal) activity (E) and quantification of IL-8 staining by IF in 4 old cell lines treated with sEVs from young or old donors (F). Representative images from AG16086 are shown. t test analysis was performed. ∗∗p < 0.01; ns, non-significant. (G) Schematic representation of the transmission of the rejuvenation potential of sEVs. Primary senescence are donor cells undergoing senescence by activation of H-Ras^G12V^ (iRAS cells); secondary and tertiary senescence are iRAS cells treated with sEVs isolated from either iC cells (iCsEV) or iRAS cells (RASsEV). (H) qPCR to determine SASP mRNA levels of cells undergoing either primary or secondary senescence. Mean of 3 independent experiments is shown. (I) Long-term treatment of 4 young or 4 old donor cells with the indicated sEVs every 72 h. Young and old donor cells with media (FBS 10%) were used as controls. Proliferation was measured by quantifying cell number ± SEM during different days. Two-way ANOVA statistical analysis was done. ∗∗∗p < 0.001. (B, C, and E) Scale bar, 50 μm. See also Figure S1.

We hypothesize that fibroblasts derived from young donors have the ability to ameliorate senescence-related features in fibroblasts from old donors, via non-cell-autonomous mechanisms. First, we confirmed the presence of biomarkers of senescence in old versus young fibroblasts (Figure S1A) (Rapisarda et al., 2017). To address our hypothesis, we pooled the conditioned media (CM) derived from four different human primary fibroblasts isolated from young donors (GM05399, GM00969, GM05565, and GM05758; ranging 1–3 years). We next dissected the CM into soluble and vesicular fractions and investigated which fraction could ameliorate senescence. Thus, we compared the complete CM fraction, with an equivalent volume of CM divided into SF, lEV, and sEV fractions (Figure S1B), by serial ultracentrifugation as done previously with some modifications (Borghesan et al., 2019, Théry et al., 2018). Next, we individually treated four human primary fibroblast lines derived from old donors (AG16086, AG06240, AG13152, and AG13222; range 67–81 years) with all the different fractions from young and old fibroblasts. Media with EV-depleted serum was used as negative control (FBS 10%). As shown in Figures 1A and 1B, the pooled CM from young donors prevented the proliferation arrest (measured by quantifying BrdU incorporation by immunofluorescence [IF]) and the number of cells staining positive for p16^INK4A^ and p21^CIP1^, all characteristics of senescence and old donor cells (Figure S1A) (Rapisarda et al., 2017). We found the SF to have slightly less ability to ameliorate these senescence-related biomarkers in comparison with the sEV fraction, while the lEV fraction had no effect on modifying these biomarkers in old donor cells (Figures 1A–1C and S1C). Next, we characterized the EV fraction and found that sEVs and lEVs showed a different EV distribution, mean particle size, and number by performing nanoparticle tracking analysis (NTA) (Figures S1D and S1E), while sEVs presented EV-related proteins such as ALIX and TSG101 (Figure S1F) and lacked endoplasmic reticulum contaminants such as calnexin (Figure S1G). It was also observed that old donors release more sEVs and lEVs than young donors (Figure S1H). Next, we determined the proliferative capacity of old donor cells treated for 72 h with the SF and sEVs—as lEVs had no functionality—replated and counted by DAPI staining at different days. The sEVs noticeably prevented the cell-cycle arrest in old donor cells in comparison with the SF (Figure 1D) 11 days after treatment (day 8). However, it cannot be excluded that longer-term experiments would be needed to confirm this. We further looked at additional biomarkers of senescence by qPCR related to the cell cycle and the SASP in the old cell line AG16086 treated with the SF and sEVs isolated from the young cell GM05399 (Figure S1I). The heatmap shows the potential of the sEV fraction from young cells in ameliorating senescence markers characteristic of old donors. We also validated some of these mRNA in all four old donor lines treated with pooled sEVs derived from young cells (hereafter named sEV-Ys) (Figure S1J). Next, we determined the activity of senescence-associated beta galactosidase (SA-β-Gal) and expression levels of IL-8 by IF, both biomarkers of senescence. In fact, treatment of all four old donor cells with sEV-Ys decreased the percentage of cells staining positive for SA-β-Gal and IL-8 (Figures 1E and 1F). Therefore, our data show that sEV-Ys have the ability to ameliorate certain markers of senescence in old cells.

### The Effect of sEV-Ys Ameliorating Senescence-Related Biomarkers Is Transmitted

Our previous results suggest that sEV-Ys can ameliorate the senescence-associated phenotype. Next, we questioned whether these effects could be maintained in old donor fibroblasts receiving sEV-Ys. To address this question, we took advantage of early passage human primary foreskin fibroblasts (HFFF2) expressing the oncogene H-RAS^G12V^ (iRAS) in a tamoxifen (4OHT)-inducible vector, which activates senescence and its corresponding control (iC) (Borghesan et al., 2019, Rapisarda et al., 2017). HFFF2 are derived from 14- to 18-week-old human fetus and their sEVs behave as sEV-Ys. Next, we compared changes in the mRNA levels by qPCR in cells undergoing senescence, iC and iRAS (primary senescence), and in iRAS cells treated with sEVs from either iC (iCsEVs, equivalent to sEV-Ys) or iRAS (RASsEVs, equivalent to sEV-Os) (secondary senescence) (Figure 1G). The heatmap in Figure 1H shows that iRAS cells treated with iCsEVs have the ability of repress mRNA transcripts related to the SASP and also cell-cycle inhibitors generally upregulated during senescence (Figure S1K). This transmission effect is maintained through tertiary senescence measured by IF staining of different markers of senescence, although to a lesser extent (Figure S1L). We and others have described that cells undergoing senescence release a larger number of particles or sEVs as a characteristic of the SASP (Borghesan et al., 2019, Takahashi et al., 2017). In fact, we also observe a decrease in the release of sEVs during secondary senescence (Figure S1M). In addition, the proliferative advantage mediated by treatment with sEV-Ys every 72 h is maintained long term in old donor cells and in iRAS cells, although to a lesser extent than young and iC cells (Figures 1I and S1N). Altogether, our data show that sEVs derived from young cells ameliorate different biomarkers of senescence, which can be further transmitted.

### sEV-Ys Ameliorate Cellular Phenotypes Associated with Aging in HGPS-Derived Fibroblasts

**Figure 2:**
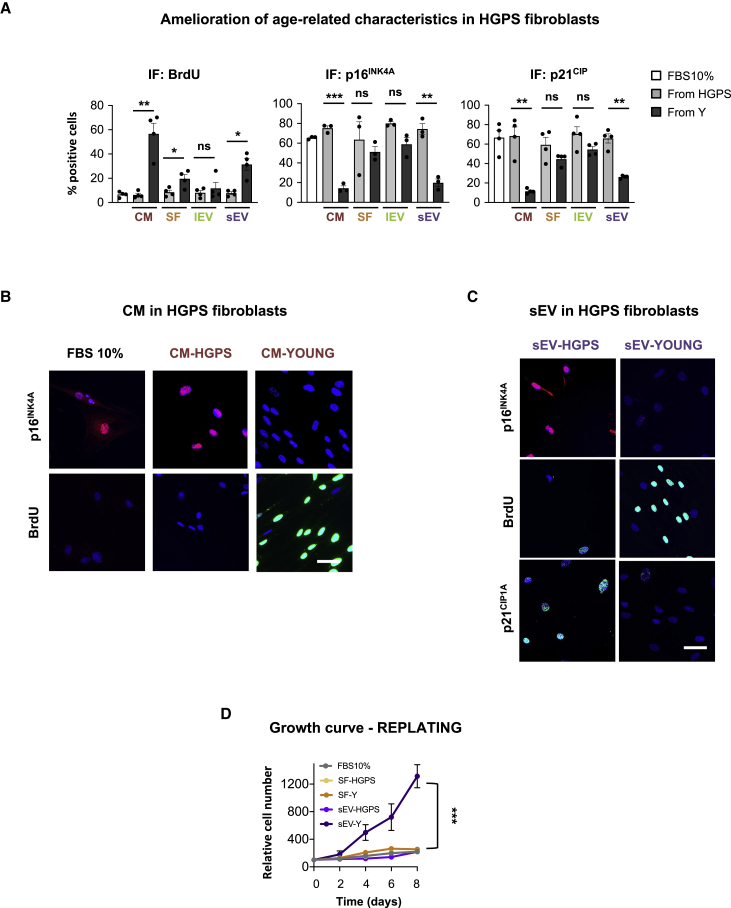
sEV-Ys Ameliorate Cellular Phenotypes Associated with Senescence in HGPS Patient-Derived Fibroblasts (A) Three or four different cells derived from HGPS patients (AG11572, AG06917, AG07493, and AG10677) were treated with pooled conditioned media (CM) and fractions (SF, lEV, and sEV) from 3–4 pooled young cells. The percentage of cells staining positive for BrdU (left), p16^INK4A^ (middle), and p21^CIP1^ (right) was quantified. Data represent the mean ± SEM of 2 independent experiments in 3–4 HGPS-derived cell lines. FBS 10% represents medium-treated cells as control. t test analyses were performed. ∗p < 0.05; ∗∗p < 0.01; ∗∗∗p < 0.001; ns, non-significant. (B and C) Representative images for AG11572 (HGPS-derived fibroblast line), treated with (B) CM or (C) sEVs derived from either HGPS or young donors. Scale bar, 50 μm. (D) Long-term growth curve of 4 HGPS-derived fibroblasts treated with either SF or sEV fractions for 72 h, replated, and counted on different days. Data show the mean ± SEM of the relative cell number along time in days. Two-way ANOVA was performed. ∗∗∗p < 0.001. See also Figure S2.

To determine if sEV-Ys within the CM had the potential to ameliorate biomarkers of aging, we took advantage of primary fibroblasts derived from patients with a severe form of premature aging, HGPS, which present biomarkers of aging and senescence (Figure S2A) (Burtner and Kennedy, 2010, Ocampo et al., 2016, Liu et al., 2011). Four different HGPS-derived fibroblasts (AG11572, AG06917, AG07493, and AG10677; ranging 2–4 years old) were treated individually with pooled CM or different fractions derived from either HGPS or young cells as in Figure 1. As shown in Figures 2A–2C, we could observe a similar pattern as with the old donors (Figure 1), where the SF and sEV fractions ameliorated the cell-cycle arrest characteristic of HGPS cells by quantifying the number of cells incorporating BrdU by IF. However, only the sEV fraction could ameliorate the high levels of expression of p16^INK4A^ and p21^CIP^ typical of HGPS cells (Figures 2A–2C), with the lEV and SF fractions having little to no effect (Figures S2B and S2C). This was further confirmed by determining by qPCR the mRNA levels of additional markers of senescence related to cell cycle and the SASP in the HGPS cell line, AG11572, with little to no effect from the SF fraction (Figure S2D). As with the sEVs derived from old donors, we could also observe an increase in sEV number from HGPS cells compared to young donors by NTA with no change in particle size (Figures S2E and S2F). Furthermore, treatment of four HGSP cells with pooled sEV-Ys prevented the proliferation arrest characteristic of these cells with no effect observed from the SF at longer-term treatment (Figure 2D).

### sEV-Ys Are Enriched in Antioxidants and Overcome the Oxidative Stress Characteristic of Old Donor Fibroblasts

**Figure 3:**
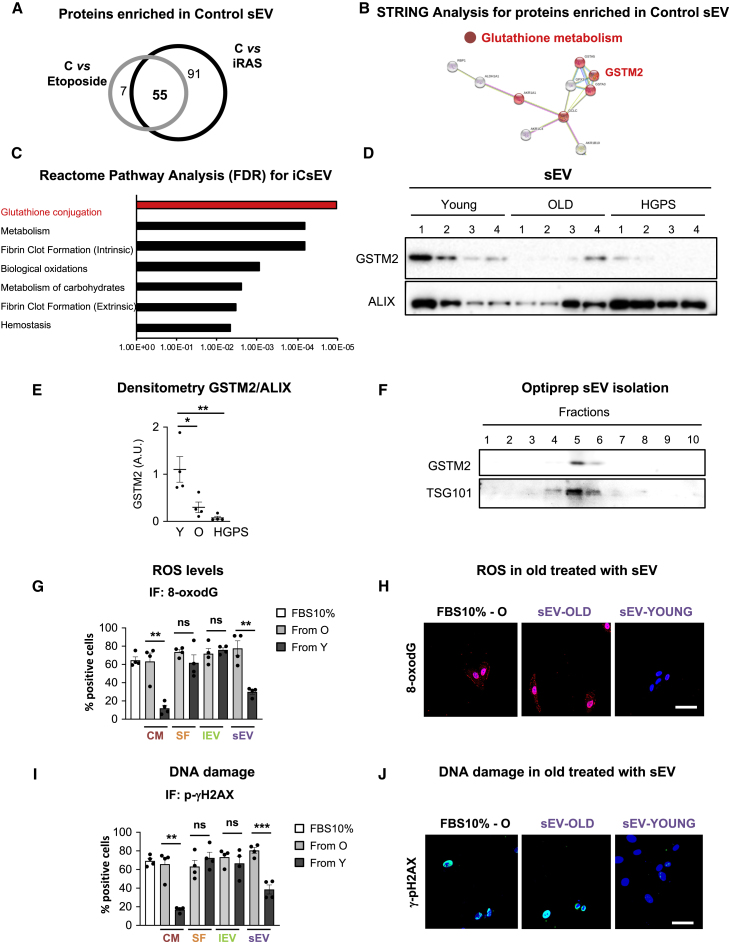
sEV-Ys Are Enriched in Proteins Related to Glutathione Conjugation and Ameliorate ROS Levels in Old Donor-Derived Fibroblasts (A) Analysis of a published proteomic analysis (Borghesan et al., 2019) on sEVs isolated from iC fibroblasts and sEVs derived from cells undergoing senescence (iRAS and by etoposide treatment). We found 55 proteins enriched in sEVs derived from iC in comparison with sEVs from iRAS and etoposide. (B and C) STRING (B) and Reactome pathway analysis (C) for the 55 proteins enriched in sEVs from iC highlight the glutathione metabolism pathway where GSTM2 is highlighted. (D) Immunoblotting for GSTM2 and ALIX in sEVs isolated from young, old, and HGPS fibroblasts loading the same number of sEVs (3 × 10^9^). sEVs isolated from all four cell lines are shown. (E) Densitometry quantification for GSTM2 immunoblotting versus ALIX. t test analyses were performed. ∗p < 0.05; ∗∗p < 0.01. (F) sEVs were subjected to density gradient by Optiprep and different fractions were immunoblotted for GSTM2 and TSG101 showing the presence of sEVs containing GSTM2. (G) IF quantification for ROS by 8-oxodG staining in 4 old fibroblasts treated with the CM and other fractions derived from young cells. t test analyses were performed. ∗∗p < 0.01; ns, non-significant. (H) Representative pictures for 8-oxodG staining in GM05565 (old cell line) treated with sEV-Ys and sEV-Os or with FBS 10%. (I) IF quantification for p-γH2AX staining in 4 old fibroblasts treated with young donor CM and fractions. t test analyses were performed. ∗∗p < 0.01; ∗∗∗p < 0.001; ns, non-significant. (J) Pictures of p-γH2AX in AG16086 (old cell line) basal levels or treated with sEV-Ys or sEV-Os. (H and J) Scale bar, 50 μm. (G and I) FBS 10% is shown as negative control. All data represent mean ± SEM in 4 different cells lines derived from old donors. See also Figure S3.

In order to determine proteins enriched in sEV-Ys that could be responsible for ameliorating senescence, we took advantage of a previous proteomics analysis performed in our lab for sEVs derived from iC, iRAS, or etoposide-treated HFFF2, which induces senescence (Borghesan et al., 2019, Rapisarda et al., 2017). We determined which proteins were enriched with statistical significance (FDR 10%) in iC in comparison with both iRAS- and etoposide-treated fibroblasts. Altogether, we identified 55 common proteins that were highly enriched in iCsEVs in comparison with both iRAS or etoposide sEVs (Figure 3A; Table S1). STRING and Reactome pathway analysis for these 55 proteins showed that some of these proteins grouped into the glutathione conjugation pathway (Figures 3B and 3C). In fact, GSTM2 is one of the proteins most highly enriched in iCsEVs in comparison with sEVs derived from iRAS and sEVs from etoposide-treated cells. Analysis of sEVs derived from young fibroblasts shows an enrichment in GSTM2 by immunoblotting in comparison with sEVs derived from old and HGPS-derived cells (Figures 3D and 3E). GSTM2 was also enriched in sEVs isolated from proliferating cells in comparison with sEVs derived from cells undergoing senescence by other triggers, such as iRAS or etoposide treatment (Figure S3A). To further assess that GSTM2 was found within the sEV fraction, we performed sEV Optiprep gradient isolation. Immunoblot analysis of different sEV fractions shows that GSTM2 floats at the same density as sEVs that contain TSG101 (Figure 3F). GSTM2 was also found to be highly expressed in young cell lysates in comparison with old and HGPS (Figure S3B). As the main activity of GST is to counteract the detrimental effects of ROS (Townsend and Tew, 2003), we next assessed the potential of sEV-Ys in reverting the accumulation of ROS in old donor cells. For this we measured 8-oxodG, a marker of oxidative stress. Quantification of old fibroblasts staining positive for 8-oxodG shows that fibroblasts treated with the CM from young cells reversed the accumulation of ROS in old cells (Figure 3G). Interestingly, we could confirm that only the sEV fraction had the potential to prevent ROS accumulation by quantifying 8-oxodG-positive cells, while the SF and lEV fractions had less effect (Figures 3G, 3H, and S3C). We next confirmed the antioxidant potential of sEV-Ys by measuring intracellular ROS with an alternative technique, H2-DCFDA measurement by fluorescence-activated cell sorting (FACS), in sEV-Y-treated old cells (Figure S3D). As an increase in ROS can lead to DNA damage, we determined the presence of p-γH2AX. As previously, treatment of old fibroblasts with the CM from young cells promoted a reduction in the percentage of cells staining positive for p-γH2AX, while no differences could be observed with the SF or the lEV fractions (Figures 3I, 3J, and S3E). Instead, the sEV fraction had a partial effect in preventing the accumulation of p-γH2AX (Figures 3I and 3J). Here, we show that GSTM2 protein expression is decreased during aging and senescence in cells and their corresponding sEVs, and that sEV-Ys are the main fraction ameliorating ROS and DNA damage levels in old donor fibroblasts in comparison with the SF.

### sEV-Ys Have Intrinsic GST Activity and Regulate GSH Levels to Mediate Rejuvenation in Old Donors

**Figure 4:**
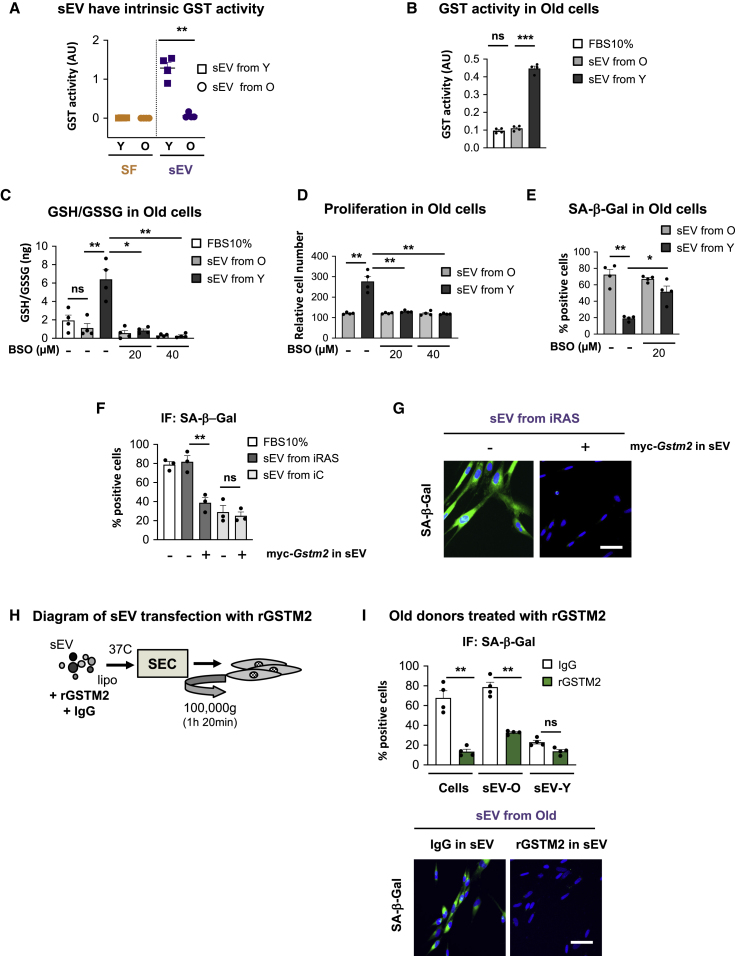
GST Activity and GSH Levels Are Important in Mediating sEV-Y Rejuvenation in Old Donors (A) sEVs isolated from 4 different young donors have independent GST activity. sEVs from old donors and their respective SF fractions from young and old donors do not present GST activity. t test analysis was performed. ∗∗p < 0.01. (B) GST activity was determined in old fibroblasts treated with sEVs from either young or old donors. FBS 10% was used as a control. Data show the mean ± SEM of 4 different donor cells. t test analysis was performed. ∗∗∗p < 0.001; ns, non-significant. (C) Ratio of GSH/GSSG in old cells treated with sEVs and different concentrations (20 or 40 μM) of BSO (buthionine sulphoximine), which prevent *de novo* GSH synthesis. The increase in GSH/GSSG levels when old cells are treated with sEV-Ys is prevented after BSO treatment. ∗p < 0.05; ∗∗p < 0.01; ns, non-significant. (D) Relative cell number shows an increase in proliferation in old cells treated with sEV-Ys, which is prevented by GSH inhibition (BSO). ∗∗p < 0.01. (E) SA-β-Gal activity downregulation by sEV-Ys is prevented by 20 μM BSO treatment. ∗p < 0.05; ∗∗p < 0.01. (F and G) iC or iRAS HFFF2 cells were treated with sEVs derived from iC or iRAS ectopically expressing myc-*Gstm2* or empty vector. The mean ± SEM from three independent experiments is shown. (F) SA-β-Gal activity was quantified and (G) representative images are shown. ∗∗p < 0.01; ns, non-significant. (H) Diagram of the protocol followed to transfect recombinant GSTM2 (rGSTM2) into old sEVs. (I) Four old donor cells were treated with sEVs isolated from old and young donors transfected with either IgG or rGSTM2 (rGSTM2-sEV). rGSTM2 was used on old donor cells on its own as a positive control. SA-β-Gal activity quantification and representative pictures are shown. Quantification represents the mean ± SEM of 4 different donor cell lines. ∗∗p < 0.01; ns, non-significant. See also Figure S4.

It has previously been reported that sEVs can have intrinsic metabolic activity (Iraci et al., 2017, Peruzzotti-Jametti et al., 2018, Yoshida et al., 2019). As our proteomic analysis and immunoblotting show that sEV-Ys have high levels of GSTM2 (Figure 3), we next set to determine whether sEV-Ys could have intrinsic GST activity compared to sEV-Os (Figure 4A). We also analyzed the SF fraction from young and old donors to determine whether soluble GST activity could be present in the SF. As shown in Figure 4A, sEV-Ys had independent metabolic activity compared to the sEV-Os, while none of the SF fractions had GST activity. We next determined whether there were changes in the GST activity in old cells treated with sEV-Ys. As we can see in Figure 4B, old recipient cells treated with sEV-Ys have an increase in GST activity in comparison with cells treated with sEV-Os or 10% FBS. Next, we set out to determine the implication of blocking the *de novo* synthesis of GSH by treating the old recipient cells with increasing concentrations (20 and 40 μM) of buthionine sulphoximine (BSO), which blocks glutamate-cysteine ligase (GCL) complex (Gorrini et al., 2013). Treatment of old donor cells with different concentrations of BSO was not toxic as no changes in cell number were observed (Figure S4A). While no effect on the ratio between reduced GSH and its oxidized form GSSG (glutathione disulfide) (GSH/GSSG) could be observed in old cells treated with sEVs from old donors, we could see that sEV-Ys induced an increase in the levels of GSH/GSSG in old cells, which was blunted when the cells were treated with different concentrations of BSO (Figure 4C). To confirm that BSO was preventing the synthesis of GSH, we treated young donor cells with different concentrations of BSO and measured the GSH/GSSG ratio (Figure S4B). Interestingly, the increase in proliferation in old cells treated with sEV-Ys and the decrease in the levels of β-Gal activity were blunted when BSO was added (Figures 4D and 4E). Altogether, these data show that sEV-Ys have intrinsic GST activity and can modulate the GSH levels in recipient cells by regulating senescence in old cells.

### GSTM2 Expression Is Partially Implicated in Preventing Senescence in Old Donor Fibroblasts

In order to determine whether GSTM2 within sEVs regulates senescence, we took advantage of a retroviral construct encoding a myc-tagged *Gstm2* construct in iC and iRAS HFFF2 cells (Dolado et al., 2007). Expression of myc-*Gstm2* in iRAS donor cells was confirmed (Figure S4C), and a partial prevention of the activation of senescence was confirmed by determining the percentage of cells expressing p16^INK4A^ and β-Gal activity (Figure S4D). The expression levels of GSTM2 in their corresponding sEVs was also confirmed (Figure S4E). Interestingly, the presence of myc-*Gstm2* within sEVs derived from iRAS cells induced a partial decrease in SA-β-Gal activity and p16^INK4A^ expression levels in iRAS cells and increased their proliferative capacity (Figures 4F, 4G, and S4F). Internalization of myc-*Gstm2* sEVs could be confirmed by immunoblot for myc tag in the recipient cells (Figure S4G). Thus, we next asked whether we could modify sEVs from old donors by transfecting a His-tagged recombinant protein for human GSTM2 (rGSTM2) as done previously (Iraci et al., 2017). Therefore, we transfected rGSTM2 or isotype IgG into sEVs isolated from old donors, and washed and re-purified these by size exclusion chromatography (SEC) followed by serial ultracentrifugation and an additional wash to avoid having excess recombinant proteins in the sEV fraction (Figure 4H). The presence of rGSTM2 within sEVs from old donors (rGSTM2-sEV) was confirmed by His immunoblotting (Figure S4H). Interestingly, we found that when we transfected rGSTM2 into sEVs from old donors and treated old cells, we prevented SA-β-Gal activity (Figure 4I). rGSTM2 on its own was used as a positive control (Figure 4I) while non-relevant recombinant proteins were used as negative controls (data not shown). Similarly, rGSTM2-sEVs downregulated the high levels of p16^INK4A^ expression and prevented the proliferation arrest characteristic of old fibroblasts (Figure S4I) and p-γH2AX (Figure S4J). Altogether, we show that GSTM2 is partially responsible for ameliorating certain biomarkers of senescence in old cells.

### sEV-Ys Ameliorate Cellular Biomarkers of Senescence *In Vivo*

**Figure 5:**
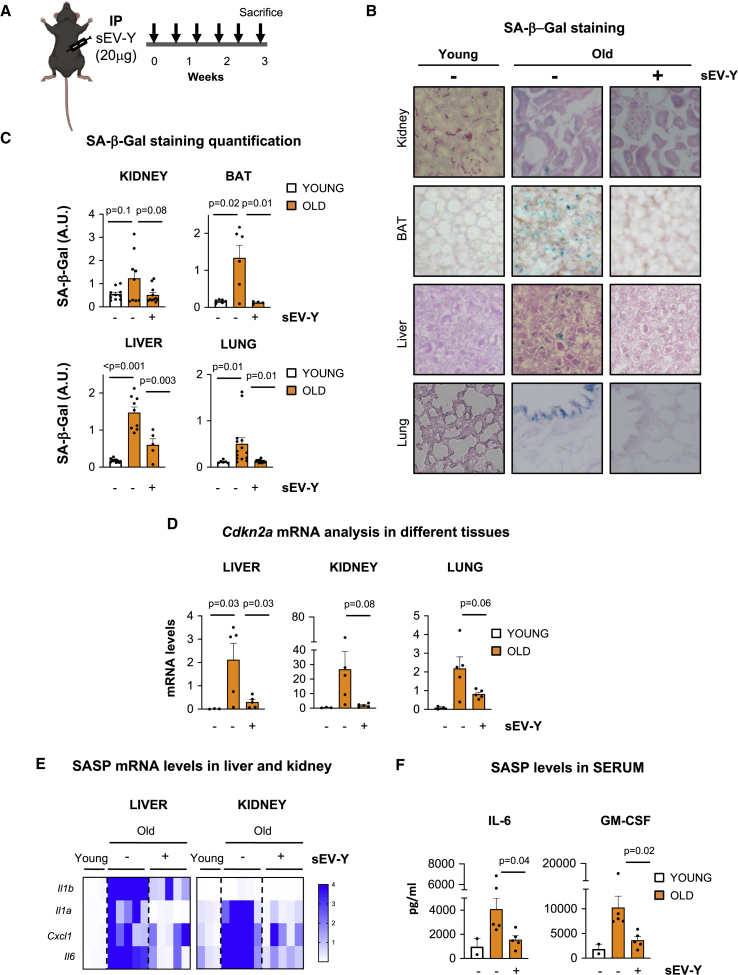
Analysis of Different Biomarkers of Aging and Senescence in Old Mice Treated with sEV-Ys (A) Schematic representation of the experimental settings for the sequential i.p. injection of 20 μg sEV-Ys twice a week for 3 weeks. Five 22- to 24-month-old mice were used per condition. (B and C) Immunohistochemistry (IHC) staining and quantification for SA-β-Gal in tissue sections from liver, kidney, lung, and brown adipose tissue (BAT) of young and old mice treated with or without sEV-Ys. Nuclear Fast Red staining is also shown. Representative pictures (B) and quantification (C) of SA-β-Gal activity for 3 young and 5 mice per condition. Welch’s t test was performed. (D) Relative *Cdkn2a* mRNA levels measured by qPCR in liver, kidney, and lung in young, old, and old mice treated with sEV-Ys. ANOVA was performed. (E) Heatmap showing mean mRNA levels for different components of the SASP in liver and kidney from young, old, and old mice treated with sEV-Ys. (F) ELISA for Il-6 and GM-CSF present in serum of the indicated mice. Data represent mean ± SEM of 2 young mice and 5 old mice per condition. Welch’s t test was performed. (D and E) Three mice were used as young controls while 5 mice were used for old and 5 for old sEV-Y-treated. See also Figure S5.

Next, we set out to evaluate if sEV-Ys could ameliorate cellular markers of aging in 22- to 25-month-old C57BL6 mice. Here, we focused on the role of sEV-Ys, as SF showed no antioxidant or GST activity *in vitro* (Figures 3 and 4). For this, we took advantage of young fibroblasts expressing an mCherry human CD63 construct to determine internalization *in vivo*. Thus, we injected 20 μg cherry-human CD63 sEV-Ys (termed sEV-Ys) by intraperitoneal (i.p.) injection or an equivalent volume of PBS twice a week for 3 weeks and determined different markers of senescence (Figure 5A). First, we evaluated indicators of senescence by SA-β-Gal staining in different tissues of treated and untreated mice. Interestingly, we could observe that sEV-Y treatment in old mice reduced the levels of SA-β-Gal staining in different tissue sections of kidney, lung, and brown adipose tissue (BAT) (Figures 5B and 5C). Furthermore, we could observe a decrease in macrophage infiltration in the liver by SA-β-Gal, suggesting a decrease in the activation of senescence in the liver as previously described (Figures 5B and 5C) (Lee and Schmitt, 2019). Quantitative mRNA analysis of *Cdkn2a* in liver, kidney, and lung showed a decreasing trend in the upregulation of *Cdkn2a* characteristic of old mice upon sEV treatment (Figure 5D). However, we could only observe a reduction in the levels of *Cdkn1a* upon treatment in the liver and kidney, not in the lung (Figure S5A). Similarly, we find a reduction in the mRNA expression of different SASP factors when old mice were treated with sEV-Ys in the liver and kidney with little to no differences in the lung (Figures 5E and S5B). Significantly, quantitative ELISA analysis of Il-6 and GM-CSF in serum, two well-characterized SASP factors (Acosta et al., 2008, Coppé et al., 2008, Kuilman et al., 2008), showed a reduction in the concentration of both SASP factors in sEV-Y-treated mice (Figure 5F). Importantly, analysis of human CD63 in the liver and kidney of mice treated with sEV-Ys (expressing human CD63), but not in the young or untreated mice, could be detected using TaqMAN probes, suggesting sEV-Y internalization (Figure S5C). HFFF2 cells were used as a positive control (Figure S5C). Interestingly, sEV-Ys induced a slight increase in brown interscapular adipose tissue mass (Figures S5D and S5E), which has been previously linked to increased lifespan (Liao et al., 2011). Therefore, we show a correlation between sEV-Y i.p. injection and a decrease in cellular biomarkers of senescence in liver, BAT, kidney, and serum, with some changes observed in the lung.

### sEV-Ys Regulate ROS and GSH Levels in Old Mice *In Vivo*

**Figure 6:**
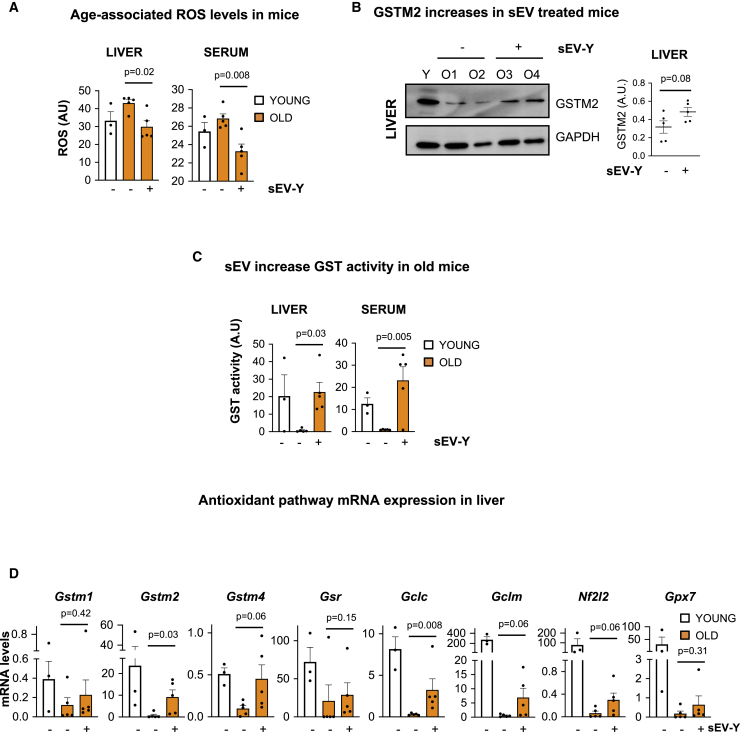
sEV-Ys Prevent ROS Accumulation and Increase the Levels of GSH in Old Mice (A) The levels of oxidative stress (ROS) were measured in liver and serum from young mice (n = 3 mice) and old mice treated or not with sEV-Ys (n = 5 old mice per condition). Data represent mean ± SEM. t test analysis was performed. (B) Representative immunoblot for the expression of GSTM2 in the liver in young and old treated or untreated mice. GAPDH was used as loading control and quantification of 5 mice per condition is shown in graph. (C) Measurement of GST activity in the liver and serum from different young, old sEV-Y-treated, or non-treated mice (n = 3 young and n = 5 per condition in old mice). Data represent mean ± SEM. t test was performed. (D) qPCR analysis for different transcripts related to the antioxidant pathway in the liver from young, old sEV-Y-treated, or untreated old mice (n = 3 young and n = 5 per condition in old mice). Mann-Whitney test was performed. See also Figure S6.

We have shown that sEV-Ys regulate ROS and GSH levels in old-derived fibroblasts (Figures 3 and 4). Next, we set to determine whether sEV-Ys could be regulating the same pathway *in vivo*. As we had seen changes in biomarkers of senescence in liver, kidney, BAT, and serum, we determined first the levels of ROS in these same tissues. Indeed, old mice presented high levels of ROS in the liver and serum compared with young mice (Figure 6A). Importantly, treatment with sEV-Ys significantly reduced the levels of ROS in these tissues, confirming the antioxidant capacity of sEV-Ys (Figure 6A), with a similar trend found in BAT and kidney (Figure S6A). As these data correlate with our *in vitro* data, we next determined if these changes in ROS could be due to a transfer of GSTM2 within sEVs, which we know is enriched in sEV-Ys. We therefore determined the levels of expression for GSTM2 in the liver by immunoblotting and found that, indeed, the levels of GSTM2 decreased in old mice but increased upon treatment with sEV-Ys (Figure 6B), with a similar trend in the kidney (Figure S6B). As these finding suggest that GST activity and GSH levels could be altered due to sEV-Y treatment, we next set out to determine whether we could observe changes in GST activity in these different tissues. In fact, GST activity was extremely decreased in the liver and serum in old mice when compared to young mice, while the treatment with sEV-Ys reverted the activity of GST to normal young levels in these tissues (Figure 6C). A similar trend was found in the kidney and BAT, although to a lesser extent in the latter (Figure S6C). Furthermore, RNA analysis for transcripts related to the glutathione antioxidant pathway confirmed changes in the transcriptome level—in particular in the master regulator *Gclc*—when old mice where treated with sEV-Ys in the liver and kidney (Figures 6D and S6D). Additionally, we set out to determine whether sEV-Ys could play a role locally by injecting 20 μg sEV-Ys in old mice subcutaneously twice a week for 2 weeks (Figure S6E). Although we looked at different biomarkers of senescence including ROS and GST activity in liver, kidney, and BAT, we could find no differences between the old mice treated with sEV-Ys or those untreated (data not shown). However, surprisingly, we did find a similar biological response as with old fibroblasts and old mice with sEV-Y i.p. injection in the serum (Figures S6F–S6H). In fact, sEV-Y treatment induced a reduction in the levels of ROS in the serum (Figure S6F) and a concomitant increase in GST activity and in the levels of reduced GSH in serum (Figures S6G and S6H). Altogether, here we show that sEV-Ys can regulate ROS and GST activity with a concurrent increase in the antioxidant GSH and related mRNA transcripts in certain tissues in old mice.

### sEV-Ys Decrease Lipid Peroxidation *In Vivo* and *In Vitro*

**Figure 7:**
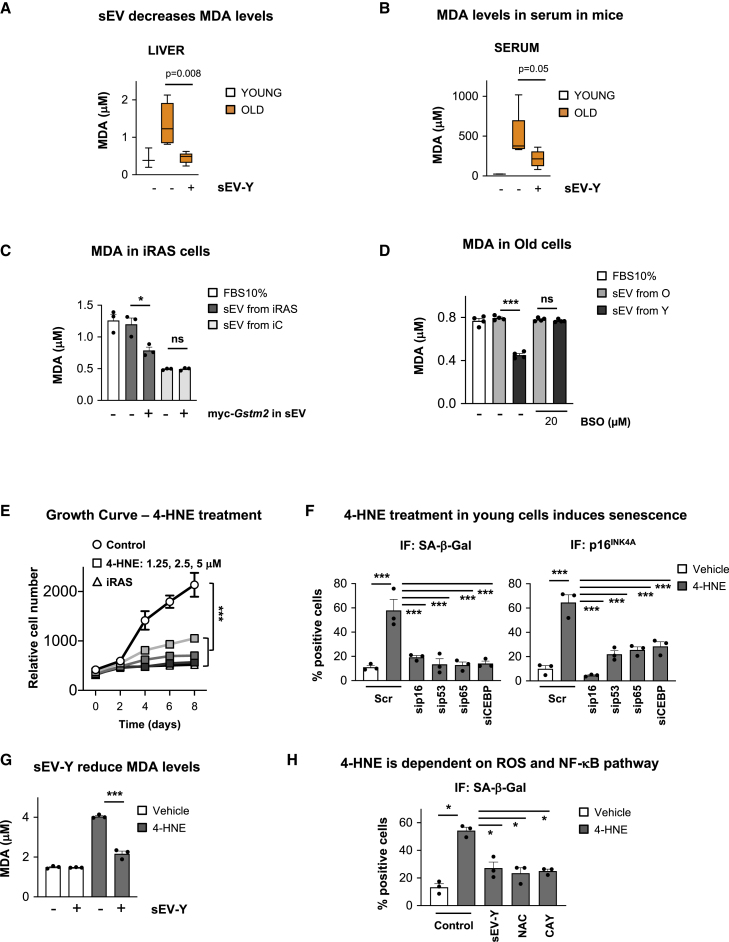
sEV-Ys Ameliorate Lipid Peroxidation *In Vitro* and *In Vivo* (A and B) MDA levels in (A) liver and (B) serum from young and old mice treated with sEV-Ys or not. Data represent the mean ± SEM of 3–5 mice. t test analysis was performed. (C) MDA quantification in iRAS cells treated with sEVs from iC or iRAS expressing myc-*Gstm2* or not. Data show the mean ± SEM of 3 independent experiments. ∗p < 0.05; ns, non-significant. (D) Quantification of MDA in old donor fibroblasts incubated with sEVs isolated from either young or old donors treated with 20 μM BSO. Graphs represents the mean ± SEM of 4 old donor fibroblasts. ∗∗∗p < 0.001; ns, non-significant. (E) Growth curve for young HFFF2 fibroblasts treated with increasing concentrations of 4-HNE (1.25, 2.5, and 5 μM). iRAS were used as a positive control. Data represent the mean ± SEM of 3 independent experiments. Two-way ANOVA was performed. ∗∗∗p < 0.001. (F) IF analyses of biomarkers of senescence (SA-β-Gal and p16^INK4A^) in 4-HNE-treated young fibroblasts with different siRNA targeting a variety of pathways that regulate senescence. 50 nM siRNA targeting p16 (sip16), p53 (sip53), NF-κB (sip65), and C/EBPβ (siCEBP) was used. Scr is a non-targeting control. ∗∗∗p < 0.001. (G) MDA levels in 4-HNE-treated young HFFF2 cells incubated or not with sEV-Ys. ∗∗∗p < 0.001. (H) Quantification of SA-β-Gal in HFFF2 cells treated with 4-HNE and different inhibitors targeting IKK (NF-κB pathway; 20 μM CAY10576) or ROS (100 nM NAC). sEV-Ys were used as a positive control. ∗p < 0.05. See also Figure S7.

As lipid peroxidation products are main endogenous substrates of GSTM2 (Townsend and Tew, 2003), we next investigated whether we could observe changes using the i.p. sEV-Y treatment in old mice. For this, we determined the levels of malondialdehyde (MDA) and 4-hydroxynonenal (4-HNE), widely used markers of lipid peroxidation (Binder et al., 2016). We could indeed observe significant changes in MDA levels in the liver and serum between young and old mice showing an increase in lipid peroxidation in old mice (Figures 7A and 7B). In accordance with our previous results, treatment of old mice with sEV-Ys prevented the accumulation of MDA in both liver and serum (Figures 7A and 7B), with a similar trend found in BAT and kidney (Figure S7A). The decrease in lipid peroxidation after sEV-Y treatment was further confirmed by 4-HNE immunoblotting in the liver and BAT (Figures S7B and S7C) and immunohistochemistry staining for 4-HNE in the kidney (Figure S7D). The reduction in lipid peroxidation due to sEV-Y treatment was also confirmed in our *in vitro* model by treating iRAS cells with sEVs isolated from iRAS expressing myc-GSTM2 and determining the levels of MDA (Figure 7C). This was further validated in old fibroblasts treated with sEV-Ys, which was blunted upon BSO treatment (Figure 7D). To further confirm an implication of sEV-Ys in ameliorating the levels of lipid peroxidation, we next treated young HFFF2 cells with increasing concentrations of 4-HNE (1.25, 2.5, and 5 μM), which induced senescence as shown by the arrest in proliferation (Figure 7E) and the increase in SA-β-Gal and p16^INK4A^ staining by IF (Figure 7F). In fact, incubation of 4-HNE-treated young HFFF2 with sEV-Ys ameliorated the increase in lipid peroxidation as shown by measuring MDA concentration (Figure 7G). Altogether, these data show that sEV-Ys have the ability to reduce the levels of lipid peroxidation *in vitro* and *in vivo*.

### Lipid Peroxidation Induces Senescence via Several Pathways

In order to determine the pathway by which 4-HNE mediates senescence, we next evaluated the effect of small interfering RNA (siRNA) and small molecule inhibitors blocking important pathways in senescence in 4-HNE-treated young fibroblasts. siRNA targeting p16 (sip16), p53 (sip53), p65—a key component of the NF-κB pathway—(sip65), and C/EBPβ (siCEBP), in addition to a non-targeting control (Scr), were used. As shown in Figure S7E, we could observe an increase in the percentage of cells expressing p16^INK4A^, p53, p65, and C/EBPβ upon 4-HNE treatment with all siRNA inducing a reduction in their respective protein levels measured by IF and quantification. Interestingly, the induction of senescence mediated by 4-HNE was dependent on all pathways analyzed (Figure 7F), where a reduction in the levels of MDA were confirmed (Figure S7F), verifying the widespread effect of lipid peroxidation. Importantly, treatment with sEV-Ys prevented the accumulation of MDA induced by 4-HNE treatment (Figure 7G). The implication of NF-κB and oxidative stress in preventing 4-HNE senescence was further confirmed by the use of different small molecule inhibitors targeting IKK activity (NF-κB pathway with 20 μM CAY10576) and ROS (100 nM NAC), where sEV-Ys were used as a positive control (Figure 7H). A decrease in the levels of MDA using these inhibitors and the implication of a DNA-damage response by analyzing the levels of p-γH2AX were further confirmed (Figures S7G and S7H).

## Discussion

A few decades ago, the notion of rejuvenation or amelioration of aging seemed unfeasible. However, in the last decades, the concept of parabiosis re-emerging and the rejuvenating cellular and tissue plasticity acquired by induced pluripotent stem cells have changed our views on the subject (Papapetrou, 2016, Scudellari, 2015). However, although some individual factors such as Notch/TGFβ signaling, oxytocin, GDF11, and MANF have been identified (Carlson et al., 2008, Elabd et al., 2014, Loffredo et al., 2013, Sousa-Victor et al., 2019), there are still many unanswered questions.

Interestingly, we previously found that sEVs isolated from senescent cells induce paracrine senescence in proliferating cells, which we termed evSASP (Borghesan et al., 2019, Fafian-Labora and O’Loghlen, 2020). In this study, we are describing that sEV-Ys ameliorate senescence in old recipient cells and old mice. Thus, there seems to be a crosstalk between both cells types via EVs; EVs inducing senescence in young cells and EVs preventing senescence in old cells. We believe this situation is what really happens *in vivo*. It is known that the tissue holds a mixture of senescent and proliferating cells. We believe that the predominance of functionality between sEV-Ys and sEV-Os will depend on the proportion of each cell present in the tissue. When the majority of cells existing in the tissue are senescent cells, the tissue homeostasis becomes compromised as there is transmission of paracrine senescence (Borghesan et al., 2019, Acosta et al., 2013); however, during the earlier stages of aging or during tissue damage, when there are still plenty of proliferating cells, these can “repair” tissue dysfunction by ameliorating the senescent phenotype of damaged cells through soluble factors (Mosteiro et al., 2016, Ocampo et al., 2016) and via sEVs as shown in this study. In fact, the temporary presence of senescent cells is generally beneficial for an individual while the accumulation is detrimental. Although there is also a huge contribution from the immune system in the clearance of senescent cells to improve tissue function, EVs also play a key role in maintaining tissue homeostasis (Kalluri and LeBleu, 2020, Lee and Schmitt, 2019, van Niel et al., 2018). It is therefore feasible that there is a simultaneous contribution of a variety of mechanisms to keep the tissue homeostasis.

We recently showed that both SF and sEVs can mediate paracrine senescence, yet lEVs had no functionality (Borghesan et al., 2019). However, when looking at ameliorating features of senescence and aging, especially in relation to oxidative stress, we find that only the sEV fraction is functional. One possible explanation could be that the SF is immediately oxidized due to the high levels of ROS present in cells from old donors and in old mice, while sEV contents are protected due to their membrane layer. This would allow sEVs to be functional both locally and systemically, as we have seen in the liver, kidney, BAT, and serum, with sEV-Ys delivered by i.p. injections, but also the serum during local sEV-Y injections. This is important and highlights the potential of sEVs as anti-aging therapeutic agents. In fact, there are several clinical trials evaluating the potential of sEVs as therapeutic agents in a variety of contexts (Fuster-Matanzo et al., 2015).

GSTM2 is a cytosolic enzyme that catalyzes the conjugation of GSH aimed at the elimination of ROS-generated toxic byproducts (Townsend and Tew, 2003). Here, we show that the levels of GSTM2 decrease during senescence and in aged mice in accordance with a decrease in its presence in their respective sEVs. Interestingly, we show that GSTM2 is contained within sEVs, conferring upon them GST antioxidant activity, which ameliorates senescence, in contrast to the SF fraction. As GSTM2-contained sEVs are a limiting factor, we observe they are the initial trigger of the antioxidant pathway, which needs to be maintained transcriptionally to see the antioxidant long-term response we observe *in vivo*. GST needs the supply of reduced GSH, which we have shown to be extremely low in old cells and mice tissue. Thus, we observe increased transcriptional expression of several GSH synthesis enzymes upon sEV-Y treatment, in particular the GSH master regulator *Gclc* boosting the intracellular GSH pool. Furthermore, our data are consistent with other studies that show that EVs can have independent metabolic activity. EVs have been shown to have asparaginase and succinase activity to suppress neuroinflammation (Iraci et al., 2017, Peruzzotti-Jametti et al., 2018), while nicotinamide phosphoribosyltransferase (eNAMPT) was shown to be present within EVs to ameliorate aging. In fact, eNAMPT-EVs can induce NAD^+^ in recipient cells and counteract aging (Yoshida et al., 2019). In spite of all the evidence, we cannot exclude the possibility that additional components within sEV-Ys are also contributing to prevent the senescent and aging phenotype observed.

One of the main substrates of GSTM2 are lipids. The oxidation of lipids induces degradation products through a process known as lipid peroxidation. This results in the generation of highly reactive products such as reactive aldehydes 4-hydroxynonenal (4-HNE) and malondialdehyde (MDA), which can modify protein function (Binder et al., 2016). It is interesting that sEV-Ys reduce 4-HNE and MDA levels in the liver, kidney, BAT, and serum in old mice, although accumulation of 4-HNE has been previously reported in senescence, aging, and age-related diseases (Ademowo et al., 2017, Jurk et al., 2014). In fact, treatment of young cells with 4-HNE induces senescence, which is prevented by treatment with sEV-Ys. The importance of senescence mediated by 4-HNE is highlighted by the use of siRNA and small molecule inhibitors targeting several pathways important in senescence.

Although it is tempting to speculate that according to our results sEV-Ys have rejuvenating potential to young tissues in old mice, we must be cautious to reach such conclusions as more experimental data would be needed. However, we cannot deny that sEV-Ys are helping damaged tissues to repair, which is also a very attractive tool. It would be interesting to perform longer-term experiments to determine the time period by which sEV-Ys can have rejuvenating or repairing functions.

### Limitations of Study

One caveat of the current study is the use of PBS as a control in old mice in comparison with sEV-Ys injected into old mice. We are aware that the ideal control would have been the use of sEV-Os in old mice as per the *in vitro* data. We hypothesized, based on our previous findings (Borghesan et al., 2019), that sEV-Os might induce paracrine senescence in the few proliferating cells that old mice have.

## STAR★Methods

### Key Resources Table

**Table:** 

REAGENT or RESOURCE	SOURCE	IDENTIFIER
**Antibodies**		

p16^INK4A^	Abcam	Cat# ab108349; RRID: AB_10858268
β-Actin	Abcam	Cat# ab8226; RRID: AB_306371
p21^CIP^	Abcam	Cat# ab109520; RRID: AB_10860537
phospho-γH2AX	Merck Millipore	Cat# 05-636-I; RRID: AB_2755003
TSG101	Abcam	Cat# ab30871; RRID: AB_2208084
ALIX	Abcam	Cat# ab88743; RRID: AB_2042597
IL-8	R&D Systems	Cat# MAB208; RRID: AB_2249110
Calnexin	Abcam	Cat# ab22595; RRID: AB_2069006
8-oxoG	Merck Millipore	Cat# MAB3560; RRID: AB_11210698
GSTM2	Sta Cruz Biotech	Cat# sc-376486; RRID: AB_11150128
GSTM2	Abcam	Cat# ab175282; RRID: AB_175282
4-HNE	Abcam	Cat# ab46545; RRID: AB_722490
Myc tag	Cell Signaling Technology	Cat # 2276S; RRID: AB_331783
BrdU	Abcam	Cat # ab6326; RRID: AB_305426
C/EBPβ	Abcam	Cat # ab15049; RRID: AB_301597
p65	Abcam	Cat# D14E12; RRID: AB_10859369

**Chemicals, Peptides, and Recombinant Proteins**		

GSTM2 Protein, Human, Recombinant (His Tag)	Sino Biological	12042-H07E
L-Buthionine-sulfoximine	Sigma-Aldrich	B2515
5,5′ dithio-bis-2-nitrobenzoic acid	Sigma-Aldrich	D8130
NADPH	Sigma-Aldrich	CAS No. 2646-71-1
Glutathione reductase	Roche	10105678001
2-vinylpyridine	Sigma-Aldrich	CAS No. 100-69-6
2’, 7’ -dichlorodihydrofluorescein diacetate	Invitrogen	D399

**Critical Commercial Assays**		

Immunofluorescence SA-β-Gal	Sigma-Aldrich	F2756
SA-β-Gal for tissue sections	Canvax	CA090
Glutathione S-Transferase (GST) Assay	Sigma-Aldrich	CS0410-1KT
Lipid peroxidation assay Colorimetric and Fluorescence	Abcam	ab118970
GSH/GSSG-Glo Assay	Promega Corporation	V6611
Thiobarbituric Acid Reactive Substances (TBARS) Assay	Canvax	CA995
4-Hydroxynonenal	Cambridge-biosciences	2083-1

**Deposited Data**		

Raw data	This paper	https://doi.org/10.17632/3kjy2vd2md.1

**Experimental Models: Cell Lines**		

HFFF2	Culture Collections (Public Health England, UK)	RRID: CVCL_2489
HEK293T	Acosta et al., 2008	Acosta et al., 2008
Young-derived primary human fibroblasts	Coriell Cell Repository	GM05399, GM00969, GM05565, GM05758
Old-derived primary human fibroblasts	Coriell Cell Repository	AG16086, AG06240, AG13152, AG13222
HGPS-derived primary human fibroblasts	Coriell Cell Repository	AG11572, AG06917, AG07493, AG10677

**Experimental Models: Organisms/Strains**		

C57BL/6J mice	Charles River	Charles River

**Oligonucleotides**		

See Table S2 for primers	This paper	Table S2
siRNA: C/EBPβ	Horizon Discovery/Dharmacon	L-006423-00-0005
siRNA: p16	QIAGEN	SI02623747
siRNA: p53	QIAGEN	SI02664403
siRNA: p65§10	QIAGEN	SI04437062

**Recombinant DNA**		

ER:RAS^G12V^	Rapisarda et al., 2017	Rapisarda et al., 2017
pWZL c-myc-*Gstm2*	Dolado et al., 2007	Dolado et al., 2007

**Software and Algorithms**		

STRING: functional protein association networks	STRING	https://string-db.org
GraphPad Prism 8	GraphPad Software	https://www.graphpad.com/scientific-software/prism/

**Other**		

NTA Calibration Beads (100 nm)	Polyscience	24041
10k protein concentration columns	EMD Millipore	10088753
qEV columns	IZON	SP5
ELISA IL-6	Mab Tag GmbH	mIL-6-EIA-1
ELISA GM-CSF	Mab Tag GmbH	mGMCSF-EIA-1

### Resource Availability

#### Lead Contact

Further information and requests for resources and reagents should be directed to and will be fulfilled by the Lead Contact, Ana O’Loghlen (a.ologhlen@qmul.ac.uk).

#### Materials Availability

No new unique reagents were generated from this study.

#### Data and Code Availability

The main data from this study has been deposited in Mendeley with the identification number https://doi.org/10.17632/3kjy2vd2md.1.

### Experimental Model and Subject Details

#### Human Primary Cell Cultures

HFFF2 (Human Caucasian fetal foreskin fibroblasts) were obtained by the Culture Collections (Public Health England, UK; ECACC 86031405). Young, old and progeria-derived primary human fibroblasts were obtained from the Coriell Cell Repository with the following codes: Young: GM05399 (1 year old; Y1; male), GM00969 (2 years old; Y2; female), GM05565 (3 years old; Y3; male), GM05758 (1 year old; Y4; male); Old: AG16086 (67 years old; O1; female), AG06240 (80 years old; O2; male), AG13152 (80 years old; O3; male), AG13222 (81 years old; O4; male); Progeria: AG11572 (2 years old; HGPS1; female), AG06917 (3 years old; HGPS2; male), AG07493 (2 years old; HGPS3; female), AG10677 (4 years old; HGPS4; male). Cells were maintained in high-glucose, pyruvate, Dulbecco’s modified Eagle’s medium with 10% fetal bovine serum and 1% antibiotic-antimycotic solution in a 37C incubator with 5% CO_2_.

#### Mice

C57BL/6 WT mice were purchased from Charles River. Sex-matched male mice between 10-12 weeks of age were used as young mice and between 22-25 months were used for old mice. Mice were housed at 20-24C, 45%–64% humidity, and at a 12-h light/dark cycle. Experimental mice were fed Harlan Teklad pellets 2018 (18% protein). Mice were specific-pathogen free [Health screening (Full-FELASA profile) was performed annually] and maintained at the animal facility at the Hospital Ramón y Cajal of Madrid (Spain). All experiments were performed in accordance with legislation in Spain (RD 53/2013) and the European Directive (2010/63/EU) and approved by the Ethics Committee of the Ramón y Cajal Hospital, Madrid (ES-280790002001).

### Method Details

#### Cell Culture, Retroviral and Lentiviral Infections

All cells were washed twice with PBS prior to starting the experiments to remove excess FBS media and maintained in sEV-depleted FBS media for the duration of the experiment. FBS was depleted of sEV by overnight (ON) ultracentrifugation at 100,000 g at 4C (Sorvall 100SE Ultracentrifuge). The supernatant was removed and stored in 50mL falcons at 20C until required. CM was collected after 72 h incubation with cells, unless specified otherwise.

Methods used for retrovirus and lentivirus production and infection have been previously described (Acosta et al., 2008, Rapisarda et al., 2017).

#### Differential Ultracentrifugation of Several Fractions from Conditioned Medium and sEV Isolation

To isolate the different EV fractions, the protocol of differential ultracentrifugation (Théry et al., 2006, Théry et al., 2018) was modified and adapted (Borghesan et al., 2019). All cells were plated for each individual experiment and thus the conditioned media was only used once. Briefly, 1x10^6^ early passage donor cells were plated in a 10cm dish (10ml media) for each individual experiment. After 72 h we either dissected the conditioned media or isolated sEV. For the disection of the CM, whole CM (10ml for one 10cm dish) was collected by pipetting from the 10cm dish in 50 mL falcon tubes, centrifuged at low speed (2,000 g for 20min; k-factor of the rotor is 41056) to eliminate dead cells and cellular debris prior to use and split in two. One half was used as whole CM, concentrated and used to treat 12 wells of a 96-well plate while the other half was further processed. From the second half, large EV (lEV) were collected after the 10,000 g centrifugation step for 1 h, washed in 15 mL PBS and spun down again at 10,000 g (k-factor of the rotor is 669) for 1 h. The soluble fraction (SF) was then filtered through a 0.22μm filter prior to the 100,000 g centrifugation step. The SF was collected after a 1 h and 20min 100,000 g centrifugation step and concentrated using a 10K column (Amicon Ultra-0.5 Filter) at 14,000 g for 10min obtaining a concentration factor 10X. The final 100,000 g pellet (sEV) was washed once in 15ml of PBS and resuspended in 10% EV-depleted FBS media for the functional cell culture experiments. Each SF, lEV and sEV individual fraction was used to treat 4 wells of a 96-well plate. In the case of sEV isolation, we resuspend the final 100,000 g pellet in 840 μl EV-depleted media and treated 12 wells of a 96-well plate (plated with 1,500 recipient cells each) with 70μl of the sEV resuspension. Thus, with 10ml of conditioned media from one 10cm dish (1x10^6^ cells) we treated 12 wells of a 96 well plate. Media with EV-depleted serum (FBS10%) was used as negative control in most experiments. A Sorvall 100SE Ultra Centrifuge, with a Beckmann Fixed Angle T865 rotor was used for sEV isolations. The k-factor of the rotor is 2,08.

We have submitted all relevant data of our experiments to the EV-TRACK knowledgebase (EV-TRACK ID: EV200036) (Van Deun et al., 2017).

#### Density Gradient sEV Isolation

sEV isolated by serial ultracentrifugation were re-suspended in 1.5 mL of suspension buffer (0.25M sucrose, 10mM Tris pH 8.0 and 1mM EDTA (pH 7.4). They were mixed 1:1 with 60% stock solution of iodixanol/Optiprep. Then, 1.4ml 40% iodixanol, 1.3ml 20% iodixanol and 1.2ml 10% iodixanol were successively layered on top of the sEV suspension and tubes were centrifuged at 100,000 g overnight, stopping without the break on. After centrifugation, ten fractions of 700μl were collected from the top of the tube. Fractions were pelleted at 100,000 g for 1 h 20 min and washed with 15 mL PBS at 100,000 g for 1 h 20 min. Finally, they were re-suspended in 50 μl lysis buffer for immunoblotting analysis.

#### qPCR Analysis

RNA was isolated using TRIzol Reagent from the cells previously washed with PBS. Tissues were stored in TRIzol and frozen until further use. cDNA was generated using the High-Capacity cDNA Reverse Transcription Kit according the manufacturerś instructions. qPCR was performed using SYBR Green PCR Master Mix on a 7500 Fast System RealTime PCR cycler. For Taqman probes, Taqman Universal Mix was used to detect the presence of human CD63 (Hs01041238_g1) in kidney and liver. GAPDH mouse and human (Mm99999915_g1 and Hs0276624_g1 respectively) were used as housekeeping genes. Primer sequences are listed in Table S2. The relative expression was calculated using the ΔΔCt methods using the Ct values generated using the 7500 software version 2.0.6. The data were normalized to a housekeeping gene, *RPS14*, except for the Taqman probes.

#### Nanoparticle Tracking Analysis (NTA)

The NanoSight LM10 (Malvern Instruments) was calibrated using Silica Microspheres beads. EVs were diluted in PBS in order to obtain a particle number between 10^8^ –10^9^ particles. Three measurements of 60 s were taken per each sample and the mean value was used to determine particle number. The movement of each particle in the field of view was measured to generate the average displacement of each particle per unit time, which was calculated using the NTA 3.0 software.

#### β-Galactosidase Staining by IF for Cells

Cells were incubated with 33 μM of the β-galactosidase substrate C12FDG (Fluorescein di-B-D-galactopyranose) in medium supplemented with 0.5% (v/v) FBS-depleted sEV for 8 h at 37 C. After the incubation, the cells were washed with PBS and fixed with 4% (v/v) paraformaldehyde for 15 min at room temperature.

#### β-Galactosidase Staining for Mouse Tissue Sections

Liver, kidney and lung were fixed using paraformaldehyde 4% (v/v) for 1 h. Tissue sections were stained with Galactosidase Assay Kit overnight at 37C. Then, the tissues were embedded in paraffin. Tissue sections from frozen BAT in OCT were incubated with Galactosidase Assay Kit overnight at 37 C.

#### Immunofluorescence Staining in Cells

The cells were washed with PBS and fixed in 4% (v/v) paraformaldehyde for 15 min at room temperature. After, the cells were washed in PBS twice and permeabilized with 0.2% (v/v) Triton X-100. Then, the cells were blocked with 1% (m/v) BSA and 0.2% (m/v) gelatin fish. Cells were incubated with the suitable concentration of primary antibody (Table S3) and for BrdU detection, the cells were incubated with 0,5 U/μl DNaseI and 3 mM MgCl_2_ overnight at 4C. The next day, the preparations were washed twice with PBS and incubated with DAPI and secondary antibody. Finally, the cells were washed with PBS twice and the IF preparation are maintained with PBS and stored at 4C until the acquisition of pictures using INCell 2200 automatized microscope.

#### High Content Analysis (HCA) Immunofluorescence in Cells

The images of immunofluorescence were acquired using the automated high throughput fluorescent microscope IN Cell Analyzer 2000 with a Å~20 objective. The fluorophores in wavelength settings were used to generate the IF images (‘DAPI’ for DAPI, ‘FITC’ for AlexaFluor 488 FITC, and ‘Cy5′ for AlexaFluor647). Multiple fields per well were acquired to include a minimum of 100 cells per sample well.

High content analysis (HCA) of the images were processed using the INCell Investigator v.2.7.3 software as described previously (Borghesan et al., 2019, Rapisarda et al., 2017). DAPI was used as a nuclear mask and a top-hat method allowed the segmentation of cells. To detect cytoplasmic staining in cultured cells, a collar of 7–9 nm around DAPI was applied. Nuclear staining in the reference wavelength, that is all the other wavelengths apart from DAPI, was quantified as an average of pixel intensity (gray scale) within the specified nuclear area. Cytoplasmic IF staining was quantified as a coefficient of variance of the pixel intensities within the collar area in the reference wavelength. In samples of cultured cells, a threshold for positive cells was assigned above the average intensity of unstained or negative control samples.

#### Immunofluorescence Staining in Tissues

Tissue sections were washed three times with PBS were permeabilized with 0.1% Triton X-100 for 15 min, then washed three times with PBS and blocked in 2% blocking buffer solution for 1 h at room temperature. Next, sections were incubated overnight with primary antibody at optimizated concentration (Table S3) in blocking buffer solution at 4°C, washed three times in PBS and incubated for 2 h with secondary antibodies diluted 1:200 in blocking buffer solution. Afterward, they were washed three times in PBS and the coverslips mounted on slides with DAPI-containing mounting solution. The acquisition of pictures was performed using a Nikon ECLIPSE Ti-e inverted microscope.

#### GST Activity

The tissues, serum, cells or sEV were homogenized in ice-cold Tris-HCl buffer (40 mM, pH 7.4). Then, the homogenized tissues were diluted 1:2 in RIPA Buffer. GST activity was determined using CDNB (S-2,4-dinitrophenyl glutathione). The homogenized tissues, cells or sEV were mixed with 20 mM L-Glutathione reduced and 10 mM CDNB in Dulbecco’s phosphate Buffered Saline (DPBS). The formation of the adduct of CDNB was monitored by measuring the rate of increase in absorbance at 405 nm for 5 min using a TECAN XFLUOR microplate reader.

#### Detection of ROS

ROS in tissues was estimated as following; tissues were homogenized in ice-cold Tris-HCl buffer (40 mM, pH 7.4) (different proportions depend on the tissue). Samples of tissue homogenate were mixed with 2’, 7’ -dichlorofluorescein diacetate (1 μM) prepared in Tris-HCl buffer (40 mM, pH 7.4). The mixture was incubated for 30 min at 37C. Finally, the fluorescence intensity of the samples was measured using a TECAN XFLUOR microplate reader (λexcitation 485 nm and λemission 525 nm).

To determine the levels of ROS in cells by FACS, the cells were incubated with DCFH 1 μM for 30 min at 37C. Then, they were washed with PBS three times. The data was acquired using NovoCyte Flow Cytometer with a 488nm laser. Gates were set using the NovoExpress Software to analyze single cells fluorescence.

#### Measure of Malondialdehyde (MDA)

Tissues were homogenized with Tris-HCl buffer (40 mM, pH 7.4). Then, the homogenized tissues were diluted with RIPA Buffer 1:2. To evaluate the levels of MDA we used the Thiobarbituric Acid Reactive Substances (TBARS) Assay. The samples were incubated with 5.3 mg/ ml thiobarbituric acid diluted in acid acetic and NaOH buffer for 1 h at 100C. Finally, the absorbance was measured using a TECAN XFLUOR microplate reader at 540 nm.

The levels of MDA in serum and cells was determined using the Lipid peroxidation assay colorimetric following the manufacturer’s instructions. The absorbance was measured at 532 nm using Synergy HT-Multi-Mode Microplate Reader.

#### Determination of GSH and GSSG

Total glutathione was extracted from the tissues using 0.4 N perchloric acid (PCA) for 30 min at 4C, and centrifuged at 12,000 rpm. Total glutathione levels were measured in 96-well plates by the addition of 5,5′ dithio-bis-2-nitrobenzoic acid (0.6 mM), NADPH (0.2 mM) and glutathione reductase (1U) and the reaction was monitored at 405 nm for 6 min using a TECAN XFLUOR microplate reader. Oxidized glutathione (GSSG) was measured as following; briefly, after PCA extraction, reduced glutathione (GSH) was derivatized with 2-vinylpyridine at room temperature for 1 h, and the reaction was monitored as above. Reduced GSH was obtained by subtracting GSSG levels from total glutathione levels. To determine the ratio GSH/GSSG in cells and serum a luminescent-based assay kit was used.

#### Isolation of Serum from Mouse Blood

2 mL of blood from mice was centrifuged at 2,000 xg for 10 min at 4C. After, the serum was isolated, aliquoted and stored at −80 C until needed.

#### ELISA

Mouse serum samples were analyzed to quantify the amount of IL6 and GM-CSF (Mab Tag GmbH) using an immune-assay ELISA. The absorbance was measure at 540 nm using Synergy HT-Multi-Mode Microplate Reader.

#### siRNA Reverse Transfection

HFFF2 fibroblast were reverse transfected with 50 nM siRNAs on a 96-well plate. After 24 h, the medium was changed. Two days later, the cells were washed and incubated with 2.5 μM 4-HNE in medium supplemented with 10% (v/v) FBS-depleted sEV and 1% (v/v) A/A for 6 days.

#### Treatments with 4-HNE

Young HFFF2 fibroblasts were treated with increasing concentrations of 4-HNE (1.25, 2.5 and 5 μM) in medium supplemented with 10% (v/v) FBS-depleted sEV and 1% (v/v) A/A for 6 days. Then, the cells were washed and treated with 100nM NAC, 100nM Vitamin C or 20 μM CAY10576.

#### Immunoblot

sEV and cells were lysed using the following lysis buffer [(Tris-HCl 20 mM pH 7.6; DTT 1 mM; EDTA 1 mM; PMSF 1 mM; benzamidine 1 mM; sodium molybdate 2 mM; b- sodium glycerophosphate 2 mM; sodium orthovanadate 0.2 mM; KCl 120 mM; 1 mg/mL (each) leupeptin, pepstatin A and antipain; NonidetTM P-40 0.5% (v/v); Triton X-100 0,1% (v/v)].

The protein from cell lysates were quantified using the Advance Protein Assay Reagent and for homogenized tissues by BCA assay kit (Thermo Fisher Scientific). Lysates were diluted with 4X Laemmli Sample Buffer and equal quantities of total protein were separated in SDS-PAGE gels, transferred to a PVDF 0.45 mm pore size membrane and probed with different antibodies (Table S2). Protein bands were detected using a SuperSignal West Pico PLUS Chemiluminescent Substrate and the ChemiDoc XRS+ System.

#### Transfection of Recombinant GSTM2 into sEV

sEV from old or young fibroblasts were isolated by ultracentrifugation and incubated with 1 μg/mL His-Tagged recombinant GSTM2 protein (rGSTM2) and 0.1 μg/μl Lipofectamine2000. sEVs were then purified using commercial SEC columns to remove excess rGSTM2, pooled together, ultracentrifuged again at 100,000 g for 1 h 20 min again and washed with 15 mL PBS. 10 ng/mL of rGSTM2 on its own was used as a positive control.

#### Mice Experiments

Male C57BL/6 mice aged 3 months for young and 22-25 month-old mice were used for this study. Intraperitoneal injections (IP) were performed every 3 days to inject either 20 μg of isolated sEV or an equivalent volume of PBS for 3 weeks. Subcutaneous injection were performed injecting 20 μg of sEV or PBS for 2 weeks. Mice were sacrificed 24 h after the last injection. Tissues were divided in sections and stored adequately according to the analysis to be performed. All procedures used in animal experiments followed Spanish (RD 53/2013) and European legislation (2010/63/EU) and were previously approved by the Ramón y Cajal Hospital Ethics Committee (Spain).

### Quantification and Statistical Analysis

#### Statistics

Statistical analysis was performed using Student’s t test except if specified otherwise in the figure legend. Results are expressed as the mean ± SD or SEM except were indicated otherwise. A p value was considered significant as the following: ^∗^ p < 0.05; ^∗∗^ p < 0.01; ^∗∗∗^ p < 0.001. Data were plotted and analyzed using GraphPad Prism 8 or Microsoft Excel 16 software.

## Supplemental Information

Document S1. Figures S1–S7 and Tables S1–S3

Document S2. Article plus Supplemental Information

## References

[bib1] Acosta J.C., O’Loghlen A., Banito A., Guijarro M.V., Augert A., Raguz S., Fumagalli M., Da Costa M., Brown C., Popov N. (2008). Chemokine signaling via the CXCR2 receptor reinforces senescence. Cell.

[bib2] Acosta J.C., Banito A., Wuestefeld T., Georgilis A., Janich P., Morton J.P., Athineos D., Kang T.W., Lasitschka F., Andrulis M. (2013). A complex secretory program orchestrated by the inflammasome controls paracrine senescence. Nat. Cell Biol..

[bib3] Ademowo O.S., Dias H.K.I., Burton D.G.A., Griffiths H.R. (2017). Lipid (per) oxidation in mitochondria: an emerging target in the ageing process?. Biogerontology.

[bib4] Basisty N., Kale A., Jeon O.H., Kuehnemann C., Payne T., Rao C., Holtz A., Shah S., Sharma V., Ferrucci L. (2020). A proteomic atlas of senescence-associated secretomes for aging biomarker development. PLoS Biol..

[bib5] Binder C.J., Papac-Milicevic N., Witztum J.L. (2016). Innate sensing of oxidation-specific epitopes in health and disease. Nat. Rev. Immunol..

[bib6] Borghesan M., Fafián-Labora J., Eleftheriadou O., Carpintero-Fernández P., Paez-Ribes M., Vizcay-Barrena G., Swisa A., Kolodkin-Gal D., Ximénez-Embún P., Lowe R. (2019). Small extracellular vesicles are key regulators of non-cell autonomous intercellular communication in senescence via the interferon protein IFITM3. Cell Rep..

[bib44] Burtner C.R., Kennedy B.K. (2010). Progeria syndromes and ageing: what is the connection?. Nat. Rev. Mol. Cell Biol..

[bib7] Carlson M.E., Hsu M., Conboy I.M. (2008). Imbalance between pSmad3 and Notch induces CDK inhibitors in old muscle stem cells. Nature.

[bib8] Conboy I.M., Conboy M.J., Wagers A.J., Girma E.R., Weissman I.L., Rando T.A. (2005). Rejuvenation of aged progenitor cells by exposure to a young systemic environment. Nature.

[bib9] Coppé J.P., Patil C.K., Rodier F., Sun Y., Muñoz D.P., Goldstein J., Nelson P.S., Desprez P.Y., Campisi J. (2008). Senescence-associated secretory phenotypes reveal cell-nonautonomous functions of oncogenic RAS and the p53 tumor suppressor. PLoS Biol..

[bib10] Coppé J.P., Desprez P.Y., Krtolica A., Campisi J. (2010). The senescence-associated secretory phenotype: the dark side of tumor suppression. Annu. Rev. Pathol..

[bib11] Dolado I., Swat A., Ajenjo N., De Vita G., Cuadrado A., Nebreda A.R. (2007). p38alpha MAP kinase as a sensor of reactive oxygen species in tumorigenesis. Cancer Cell.

[bib12] Elabd C., Cousin W., Upadhyayula P., Chen R.Y., Chooljian M.S., Li J., Kung S., Jiang K.P., Conboy I.M. (2014). Oxytocin is an age-specific circulating hormone that is necessary for muscle maintenance and regeneration. Nat. Commun..

[bib13] Fafian-Labora J., O’Loghlen A. (2020). Classical and non-classical intercellular communication in senescence and ageing. Trends Cell Biol..

[bib14] Fuster-Matanzo A., Gessler F., Leonardi T., Iraci N., Pluchino S. (2015). Acellular approaches for regenerative medicine: on the verge of clinical trials with extracellular membrane vesicles?. Stem Cell Res. Ther..

[bib15] Gorrini C., Harris I.S., Mak T.W. (2013). Modulation of oxidative stress as an anticancer strategy. Nat. Rev. Drug Discov..

[bib16] Iraci N., Gaude E., Leonardi T., Costa A.S.H., Cossetti C., Peruzzotti-Jametti L., Bernstock J.D., Saini H.K., Gelati M., Vescovi A.L. (2017). Extracellular vesicles are independent metabolic units with asparaginase activity. Nat. Chem. Biol..

[bib17] Jeon O.H., Wilson D.R., Clement C.C., Rathod S., Cherry C., Powell B., Lee Z., Khalil A.M., Green J.J., Campisi J. (2019). Senescence cell-associated extracellular vesicles serve as osteoarthritis disease and therapeutic markers. JCI Insight.

[bib18] Jurk D., Wilson C., Passos J.F., Oakley F., Correia-Melo C., Greaves L., Saretzki G., Fox C., Lawless C., Anderson R. (2014). Chronic inflammation induces telomere dysfunction and accelerates ageing in mice. Nat. Commun..

[bib19] Kalluri R., LeBleu V.S. (2020). The biology, function, and biomedical applications of exosomes. Science.

[bib20] Kuilman T., Michaloglou C., Vredeveld L.C.W., Douma S., van Doorn R., Desmet C.J., Aarden L.A., Mooi W.J., Peeper D.S. (2008). Oncogene-induced senescence relayed by an interleukin-dependent inflammatory network. Cell.

[bib21] Kuilman T., Michaloglou C., Mooi W.J., Peeper D.S. (2010). The essence of senescence. Genes Dev..

[bib22] Lee S., Schmitt C.A. (2019). The dynamic nature of senescence in cancer. Nat. Cell Biol..

[bib23] Liao C.Y., Rikke B.A., Johnson T.E., Gelfond J.A., Diaz V., Nelson J.F. (2011). Fat maintenance is a predictor of the murine lifespan response to dietary restriction. Aging Cell.

[bib24] Liu G.H., Barkho B.Z., Ruiz S., Diep D., Qu J., Yang S.L., Panopoulos A.D., Suzuki K., Kurian L., Walsh C. (2011). Recapitulation of premature ageing with iPSCs from Hutchinson-Gilford progeria syndrome. Nature.

[bib25] Loffredo F.S., Steinhauser M.L., Jay S.M., Gannon J., Pancoast J.R., Yalamanchi P., Sinha M., Dall’Osso C., Khong D., Shadrach J.L. (2013). Growth differentiation factor 11 is a circulating factor that reverses age-related cardiac hypertrophy. Cell.

[bib26] López-Otín C., Blasco M.A., Partridge L., Serrano M., Kroemer G. (2013). The hallmarks of aging. Cell.

[bib27] McHugh D., Gil J. (2018). Senescence and aging: causes, consequences, and therapeutic avenues. J. Cell Biol..

[bib28] Mosteiro L., Pantoja C., Alcazar N., Marión R.M., Chondronasiou D., Rovira M., Fernandez-Marcos P.J., Muñoz-Martin M., Blanco-Aparicio C., Pastor J. (2016). Tissue damage and senescence provide critical signals for cellular reprogramming in vivo. Science.

[bib29] Muñoz-Espín D., Serrano M. (2014). Cellular senescence: from physiology to pathology. Nat. Rev. Mol. Cell Biol..

[bib30] Ocampo A., Reddy P., Martinez-Redondo P., Platero-Luengo A., Hatanaka F., Hishida T., Li M., Lam D., Kurita M., Beyret E. (2016). In vivo amelioration of age-associated hallmarks by partial reprogramming. Cell.

[bib31] Papapetrou E.P. (2016). Induced pluripotent stem cells, past and future. Science.

[bib32] Peruzzotti-Jametti L., Bernstock J.D., Vicario N., Costa A.S.H., Kwok C.K., Leonardi T., Booty L.M., Bicci I., Balzarotti B., Volpe G. (2018). Macrophage-derived extracellular succinate licenses neural stem cells to suppress chronic neuroinflammation. Cell Stem Cell.

[bib33] Rapisarda V., Borghesan M., Miguela V., Encheva V., Snijders A.P., Lujambio A., O’Loghlen A. (2017). Integrin beta 3 regulates cellular senescence by activating the TGF-β pathway. Cell Rep..

[bib34] Scudellari M. (2015). Ageing research: blood to blood. Nature.

[bib35] Sousa-Victor P., Neves J., Cedron-Craft W., Ventura P.B., Liao C.Y., Riley R.R., Soifer I., van Bruggen N., Kolumam G.A., Villeda S.A. (2019). MANF regulates metabolic and immune homeostasis in ageing and protects against liver damage. Nat Metab.

[bib36] Takahashi A., Okada R., Nagao K., Kawamata Y., Hanyu A., Yoshimoto S., Takasugi M., Watanabe S., Kanemaki M.T., Obuse C., Hara E. (2017). Exosomes maintain cellular homeostasis by excreting harmful DNA from cells. Nat. Commun..

[bib42] Théry C., Amigorena S., Raposo G., Clayton A. (2006). Isolation and characterization of exosomes from cell culture supernatants and biological fluids. Curr. Protoc. Cell Biol..

[bib43] Théry C., Witwer K.W., Aikawa E., Alcaraz M.J., Anderson J.D., Andriantsitohaina R., Antoniou A., Arab T., Archer F., Atkin-Smith G.K. (2018). Minimal Information for Studies of Extracellular Vesicles 2018 (MISEV2018): a position statement of the International Society for Extracellular Vesicles and update of the MISEV2014 guidelines. J. Extracell. Vesicles.

[bib37] Townsend D.M., Tew K.D. (2003). The role of glutathione-S-transferase in anti-cancer drug resistance. Oncogene.

[bib38] Van Deun J., Mestdagh P., Agostinis P., Akay Ö., Anand S., Anckaert J., Martinez Z.A., Baetens T., Beghein E., Bertier L., EV-TRACK Consortium (2017). EV-TRACK: transparent reporting and centralizing knowledge in extracellular vesicle research. Nat. Methods.

[bib39] van Niel G., D’Angelo G., Raposo G. (2018). Shedding light on the cell biology of extracellular vesicles. Nat. Rev. Mol. Cell Biol..

[bib40] Villeda S.A., Plambeck K.E., Middeldorp J., Castellano J.M., Mosher K.I., Luo J., Smith L.K., Bieri G., Lin K., Berdnik D. (2014). Young blood reverses age-related impairments in cognitive function and synaptic plasticity in mice. Nat. Med..

[bib41] Yoshida M., Satoh A., Lin J.B., Mills K.F., Sasaki Y., Rensing N., Wong M., Apte R.S., Imai S.I. (2019). Extracellular vesicle-contained eNAMPT delays aging and extends lifespan in mice. Cell Metab..

