# Supplementary material for: Small Extracellular Vesicles Have GST Activity and Ameliorate Senescence-Related Tissue Damage
Source: Cell Metab. 2020 Jul 7;32(1):71–86.e5. doi: 10.1016/j.cmet.2020.06.004 (PMC7342013; doi:10.1016/j.cmet.2020.06.004)
Supplement: Document S1. Figures S1–S7 and Tables S1–S3 [file mmc1.pdf]

Cell Metabolism, Volume *32*

Supplemental Information

Small Extracellular Vesicles Have GST Activity and Ameliorate Senescence-Related Tissue Damage

Juan Antonio Fafián-Labora, Jose Antonio Rodríguez-Navarro, and Ana O'Loghlen

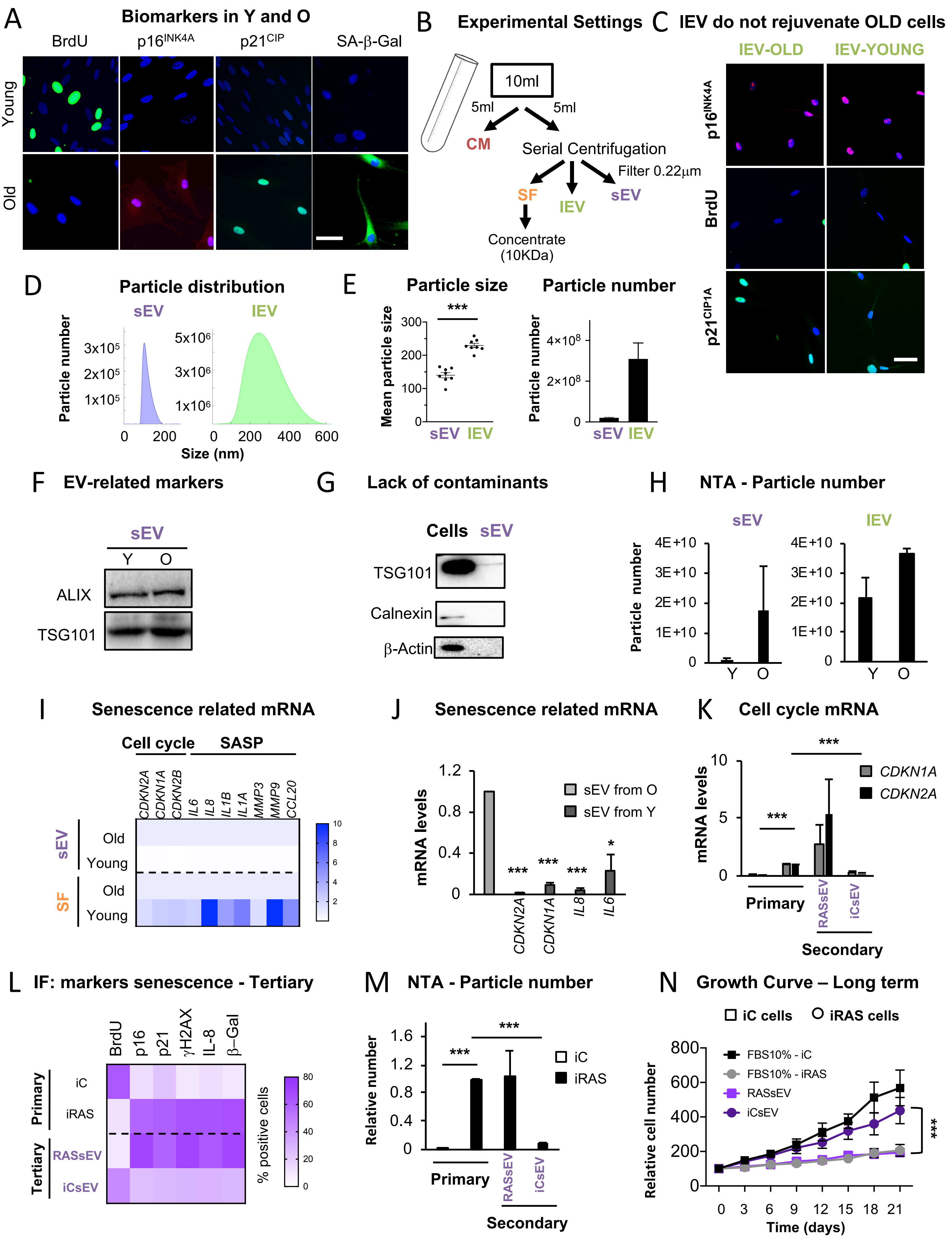
A
Biomarkers in Y and O
BrdU
p16INK4A
p21CIP
SA-β-Gal
Young
Old
B
Experimental Settings
10ml
5ml
5ml
CM
Serial Centrifugation
Filter 0.22μm
SF
lEV
sEV
Concentrate
(10KDa)
C
lEV do not rejuvenate OLD cells
lEV-OLD
lEV-YOUNG
p16INK4A
BrdU
p21CIP1A
D
Particle distribution
sEV
lEV
Particle number
3x10^5
2x10^5
1x10^5
5x10^6
3x10^6
1x10^6
0
200
400
600
Size (nm)
E
Particle size
Particle number
Mean particle size
300
200
100
0
***
sEV
lEV
4×10^8
2×10^8
F
EV-related markers
sEV
Y
O
ALIX
TSG101
G
Lack of contaminants
Cells
sEV
TSG101
Calnexin
β-Actin
H
NTA - Particle number
4E+10
3E+10
2E+10
1E+10
I
Senescence related mRNA
Cell cycle
SASP
CDKN2A
CDKN1A
CDKN2B
IL6
IL8
IL1B
IL1A
MMP3
MMP9
CCL20
Old
Young
10
8
6
4
2
J
Senescence related mRNA
mRNA levels
1.2
0.8
0.4
sEV from O
sEV from Y
*
K
Cell cycle mRNA
10
5
Primary
RASsEV
iCsEV
Secondary
L
IF: markers senescence - Tertiary
p16
p21
γH2AX
IL-8
β–Gal
Primary
Tertiary
iC
iRAS
% positive cells
80
60
40
20
M
1.6
Relative number
N
Growth Curve – Long term
iC cells
iRAS cells
FBS10% - iC
FBS10% - iRAS
Relative cell number
Time (days)
0
3
6
9
12
15
18
21

Figure S1. Analysis of soluble and vesicle fraction of young donors on old recipient cells. Related to Figure 1.

(**A**) Representative images showing a number of biomarkers of senescence such as proliferation measured by BrdU staining, $p16^{INK4A}$, $p21^{CIP}$ and senescence-associated β-galactosidase (SA- β-Gal) in young and old fibroblasts (GM05399 for Young and AG16086 for Old). Scale bar, 50 μm. (**B**) Schematic representation of the workflow followed to dissect the Conditioned Media (CM). SF, soluble factors; lEV, large extracellular vesicles; sEV, small extracellular vesicles. (**C**) Representative images of AG16086 (old cell line) treated with lEV show no changes in $p16^{INK4A}$, BrdU and $p21^{CIP}$ staining. Scale bar, 50 μm. (**D**) Representative sEV and lEV particle distribution measured by Nanoparticle Tracking Analysis (NTA). (**E**) Mean particle size and number of sEV and lEV isolated from young and old donor cells measured by NTA. T-test analysis was performed. (**F**) Immunoblot analysis for sEV-related markers ALIX and TSG101 in sEV isolated from Young (Y) and Old (O) donors. sEV were loaded by the same particle number ($3x10^9$). (**G**) Immunoblot for sEV-related marker, TSG101, and lack of endoplasmic reticulum contamination, Calnexin, in cells *versus* sEV lysates. β-Actin is used as loading control. Lysates were loaded by protein concentration. (**H**) Quantification of sEV and lEV by NTA derived from Y and O donors. Data show the mean ± SEM of sEV from 4 different young or 4 old-derived fibroblasts. (**I**) Heatmap showing relative mRNA levels related to the cell cycle and the SASP of AG16086 (Old fibroblast) treated with SF or sEV from GM05399 (Young fibroblast). 2-3 independent experiments were performed. (**J**) qPCR analysis to determine the mRNA levels of 4 different old-derived cells treated with sEV from young cells. Data represent the mean ± SEM of 4 old cells treated with sEV-Y from 1-2 biological replicates. (**K**) qPCR analysis of iRAS cells undergoing primary or secondary senescence by sEV treatment. Primary cells are donor cells while secondary are iRAS cells treated with sEV from either iC cells (iC sEV) or iRAS cells (RASsEV). Data represent the mean ± SEM of 3 independent experiments. (**L**) Heatmap showing the mean of the percentage of cells staining positive for a number of markers of senescence by IF comparing primary *versus* tertiary senescence. Data represent the mean of 3 biological replicates and indicate the percentage of cells staining positive for each antibody. (**M**) sEV particle number was measured by NTA analysis in primary and secondary senescence. Mean ± SEM of 3 experiments is shown. (**N**) Long term growth curve in iRAS cells treated every 72h with iC sEV or iC cells treated with iRAS sEV. iC and iRAS cells with FBS 10% were used as controls. Curves represent the mean ± SEM of the relative cell numbers from 3 independent experiments. Two-way ANOVA was performed.

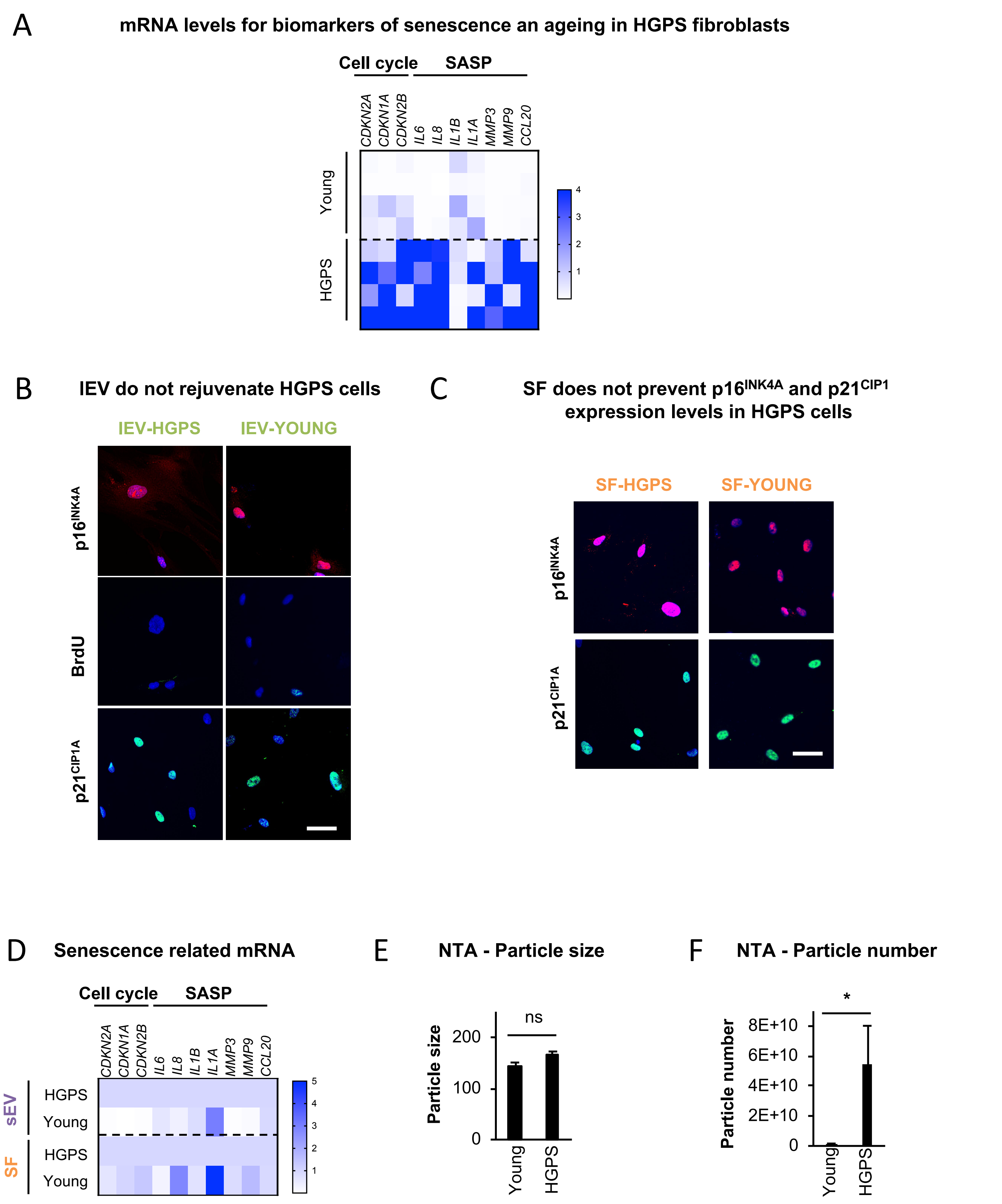
A
mRNA levels for biomarkers of senescence an ageing in HGPS fibroblasts
Cell cycle
SASP
CDKN2A
CDKN1A
CDKN2B
IL6
IL8
IL1B
IL1A
MMP3
MMP9
CCL20
Young
HGPS
B
lEV do not rejuvenate HGPS cells
lEV-HGPS
lEV-YOUNG
p16INK4A
BrdU
p21CIP1A
C
SF does not prevent p16INK4A and p21CIP1 expression levels in HGPS cells
SF-HGPS
SF-YOUNG
D
Senescence related mRNA
sEV
SF
E
NTA - Particle size
Particle size
ns
F
NTA - Particle number
Particle number
8E+10
6E+10
4E+10
2E+10
*

Figure S2. Changes of senescence markers in HGPS cells treated with sEV-Y. Related to Figure 2.
(**A**) qPCR analysis for the mRNA expression level of different markers related to senescence in four different HGPS-derived fibroblasts. Data are represented in a heatmap showing 4 individual young or 4 HGPS-derived donor cells. (**B, C**) Representative images of HGPS cell (AG11572) treated with either (**B**) lEV or (**C**) SF fractions from 3-4 pooled young cells. Scale bar, 50 μm. (**D**) qPCR analysis of HGPS cell (AG11572) treated with either sEV or SF from HGPS (AG11572) or young (GM05399) cells. Heatmap shows the mean of 2-3 independent experiments. (**E**) Particle size measurement by NTA show no changes in size between sEV derived from Young or HGPS (data represent the mean from 4 independent cell lines ± SEM). (**F**) Differences in sEV particle number isolated from Young and HGPS-fibroblasts (mean from 4 independent cell lines ± SEM).

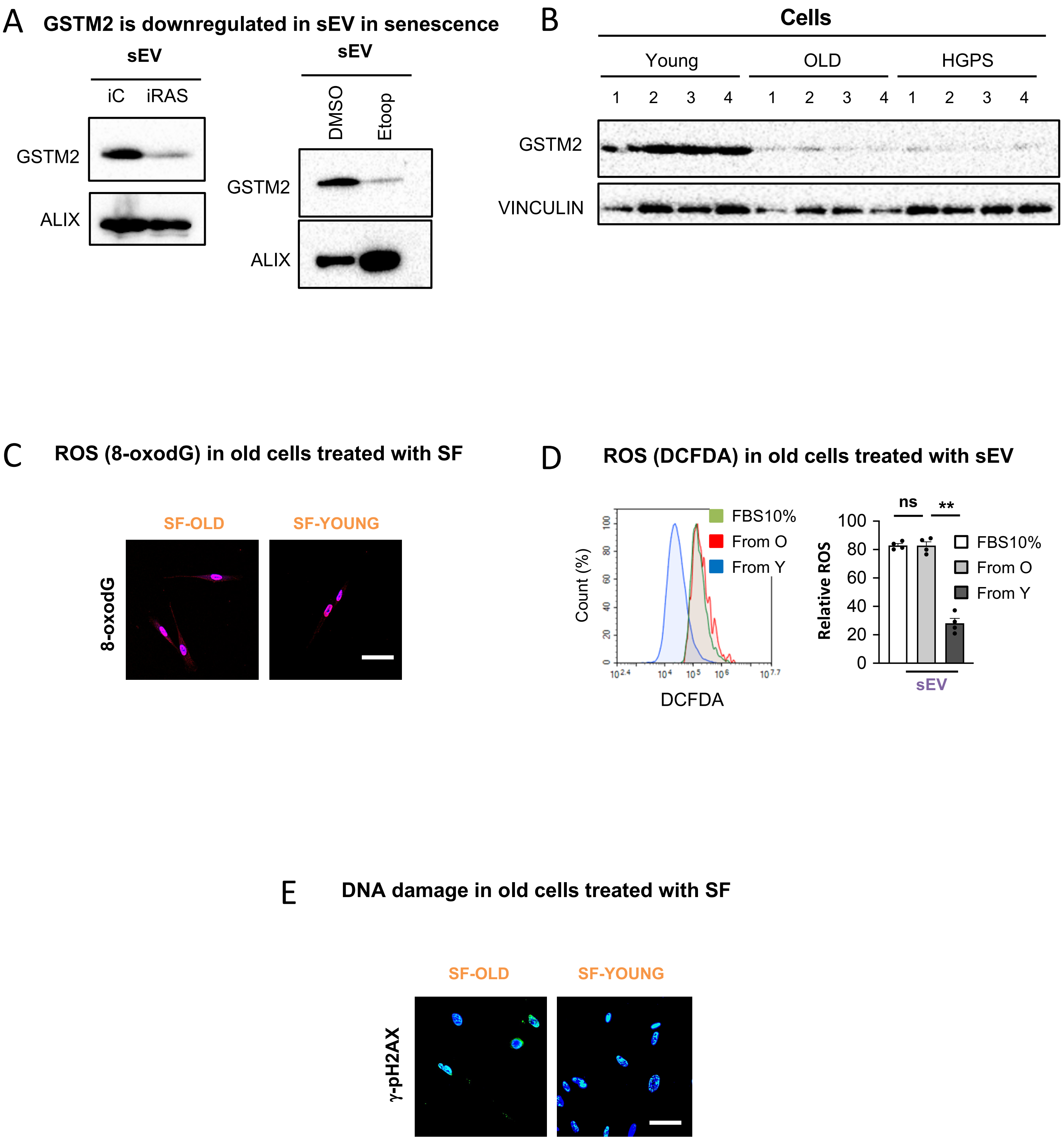
A
GSTM2 is downregulated in sEV in senescence
sEV
iC iRAS
GSTM2
ALIX
sEV
DMSO
Etoop
GSTM2
ALIX
B
Cells
Young
OLD
HGPS
1 2 3 4 1 2 3 4 1 2 3 4
GSTM2
VINCULIN
C
ROS (8-oxodG) in old cells treated with SF
SF-OLD
SF-YOUNG
8-oxodG
D
ROS (DCFDA) in old cells treated with sEV
FBS10%
From O
From Y
Count (%)
DCFDA
Relative ROS
ns
**
100
80
60
40
20
0
sEV
E
DNA damage in old cells treated with SF
SF-OLD
SF-YOUNG
γ-pH2AX

Figure S3. GSTM2 decreases in sEV isolated from senescent HFFF2 and the SF of young fibroblasts does not prevent ROS in old donors. Related to Figure 3.
(**A**) Immunoblotting for GSTM2 and ALIX in sEV derived from iC fibroblasts or fibroblasts undergoing senescence: iRAS or 50 µM Etoposide treated (Etop). Same number of sEV was loaded ($3x10^9$). (**B**) Immunoblotting for GSTM2 in cell lysates from 4 young, 4 old, and 4 HGPS derived fibroblasts. Vinculin was used as loading control. (**C**) Representative pictures of the old cell line GM05565 treated with the SF fraction from young donor cells. Staining for 8-oxodG by IF is shown. (**D**) Intracellular ROS levels were quantified by measuring DCFDA in 4 old fibroblasts cells treated with sEV-Y. Data represent the mean ± SEM of 4 cell lines. (**E**) Staining for p-γH2AX by IF is shown in the old cell line (AG16086) treated with sEV-Y. (**C, E**) Scale bar, 50 µm.

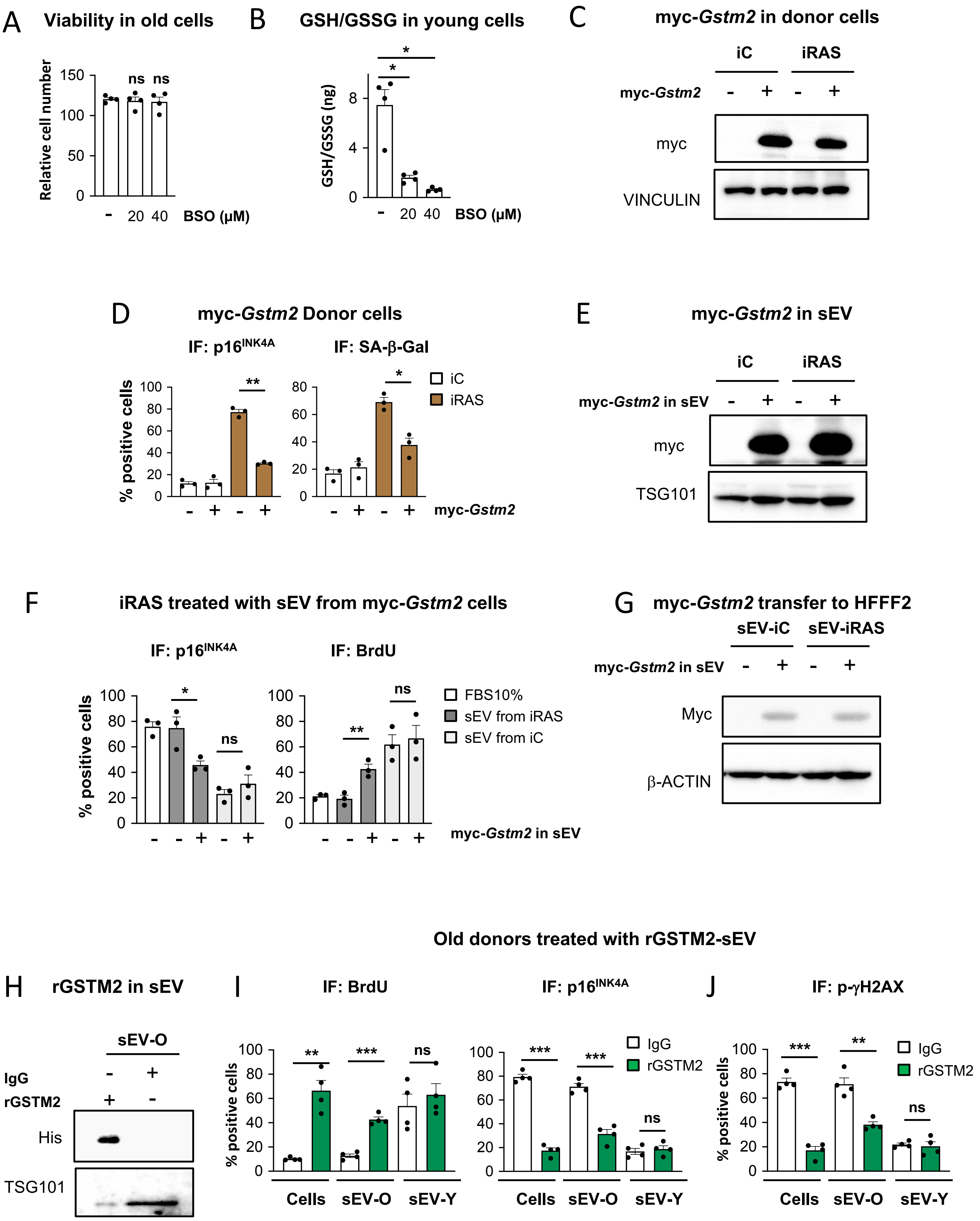
A
Viability in old cells
Relative cell number
150
100
50
0
ns ns
- 20 40 BSO (μM)
B
GSH/GSSG in young cells
GSH/GSSG (ng)
8
4
0
*
*
- 20 40 BSO (μM)
C
myc-Gstm2 in donor cells
iC iRAS
myc-Gstm2 - + - +
myc
VINCULIN
D
myc-Gstm2 Donor cells
IF: p16INK4A
IF: SA-β-Gal
% positive cells
iC
iRAS
**
*
- + - + myc-Gstm2
E
myc-Gstm2 in sEV
iC iRAS
myc-Gstm2 in sEV - + - +
myc
TSG101
F
iRAS treated with sEV from myc-Gstm2 cells
IF: p16INK4A
IF: BrdU
% positive cells
FBS10%
sEV from iRAS
sEV from iC
*
ns
**
ns
- - + - + myc-Gstm2 in sEV
G
myc-Gstm2 transfer to HFFF2
sEV-iC sEV-iRAS
myc-Gstm2 in sEV - + - +
Myc
β-ACTIN
Old donors treated with rGSTM2-sEV
H
rGSTM2 in sEV
sEV-O
IgG - +
rGSTM2 + -
His
TSG101
I
IF: BrdU
IF: p16INK4A
% positive cells
IgG
rGSTM2
** *** ns
*** *** ns
Cells sEV-O sEV-Y
J
IF: p-γH2AX
% positive cells
IgG
rGSTM2
*** ** ns
Cells sEV-O sEV-Y

Figure S4. Implication of GST in ameliorating senescence in old donors. Related to Figure 4.

(**A**) Cell number quantification in old donor fibroblasts treated with different concentrations of BSO (20 or 40 μM concentration) show no cell death. (**B**) Young donor cells present high levels of GSH/GSSG ratio which decrease significantly when the cells are treated with increasing concentrations of BSO (20 or 40 μM). (**C**) Immunoblotting for myc-tag in iC and iRAS donor cells showing the levels of expression of ectopic myc-*Gstm2*. Vinculin is used as loading control. (**D**) IF for $p16^{INK4A}$ and SA-β-Gal activity in iC and iRAS cells expressing empty vector or myc-*Gstm2* donor cells. (**E**) myc-*Gstm2* expression in sEV derived from iC or iRAS cells is detected using a myc-tag antibody. TSG101 is used as loading control. (**F**) BrdU and $p16^{INK4A}$ quantification in iRAS cells treated with sEV expressing an empty vector or myc-*Gstm2* in iC or iRAS cells. (**G**) Myc expression detected using a myc-tag antibody in recipient cells treated with sEV containing myc-*Gstm2* or not confirm sEV transfer into recipient cells. (**H**) Immunoblotting for His in sEV isolated from old donor cells transfected with either IgG or His-rGSTM2. (**I**) Proliferation (BrdU) and $p16^{INK4A}$ protein levels were measured by quantifying BrdU incorporation and $p16^{INK4A}$ by IF in old cells treated with rGSTM2-sEV from old or young sEV. rGSTM2 was used on old donor cells on its own as a positive control. (**J**) p-γH2AX levels in old cells treated with rGSTM2-sEV.

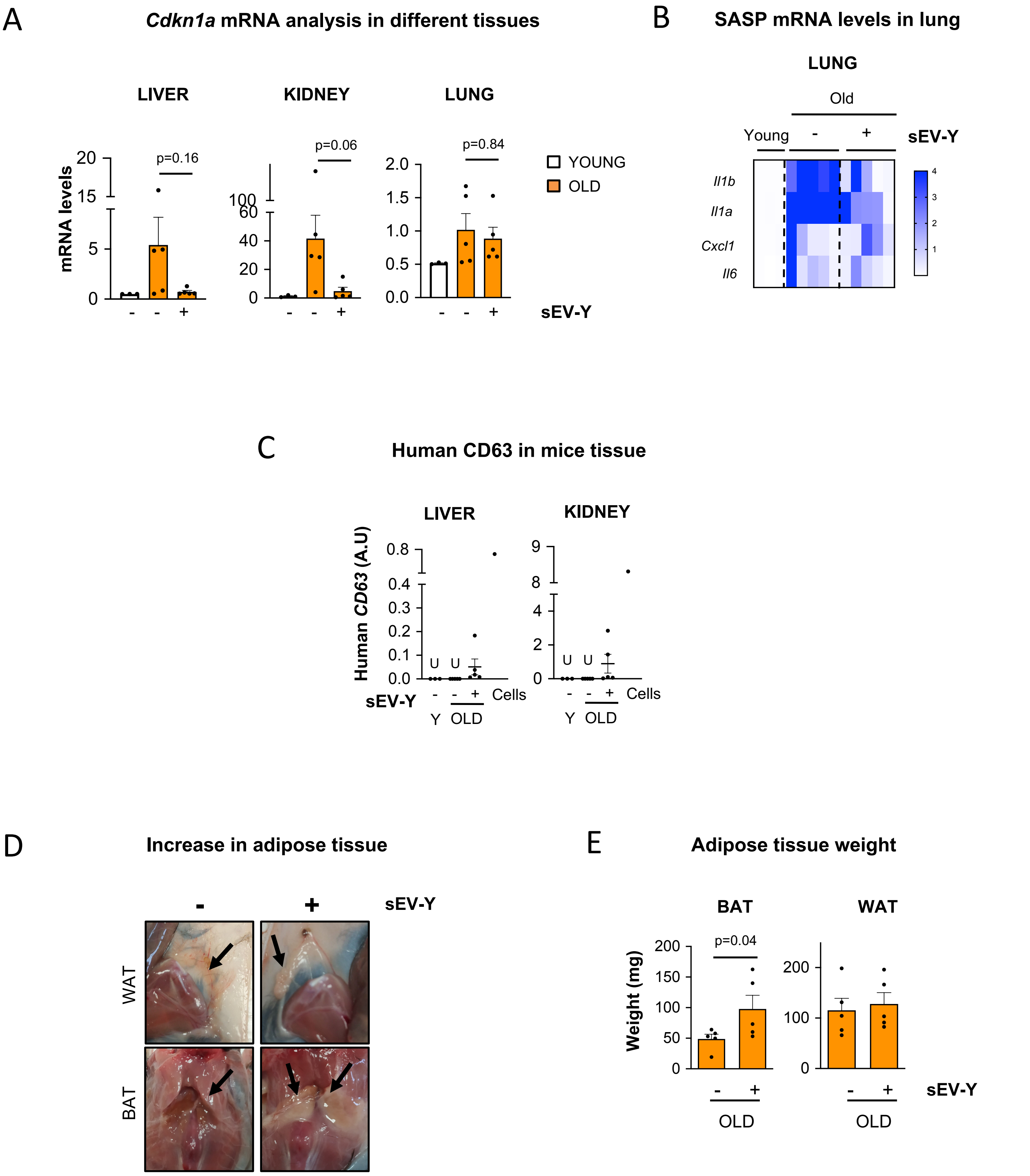
A
Cdkn1a mRNA analysis in different tissues
LIVER
KIDNEY
LUNG
mRNA levels
p=0.16
p=0.06
p=0.84
YOUNG
OLD
sEV-Y
B
SASP mRNA levels in lung
LUNG
Old
Young
sEV-Y
Il1b
Il1a
Cxcl1
Il6
C
Human CD63 in mice tissue
LIVER
KIDNEY
Human *CD63* (A.U)
U
Cells
sEV-Y
Y
OLD
D
Increase in adipose tissue
sEV-Y
WAT
BAT
E
Adipose tissue weight
BAT
WAT
Weight (mg)
p=0.04
sEV-Y
OLD

Figure S5. sEV-Y induce a slight increase in adipose tissue mass. Related to Figure 5.

(**A**) Quantitative *Cdkn1a* mRNA levels in liver, kidney and lung from young, old sEV-Y treated and untreated mice. Data show the mean ± SEM of 3 young and 5 old mice per condition. (**B**) qPCR to determine the mRNA levels of different SASP components in lung from young and old mice treated with or without sEV-Y. Data show the mean of 3-5 mice and are represented in a heatmap. (**C**) Quantification of relative human CD63 expression normalized to mouse GAPDH using TaqMAN probes in young and old mice where Ct values where undetermined (U). An increase in expression levels could be determined in the liver and kidney of 5 old mice treated with sEV-Y expressing human CD63. Normal HFFF2 cells were used as positive control where human CD63 is normalized to human GAPDH. (**D**) Representative pictures of brown interscapular adipose tissue (BAT) and white subcutaneous inguinal adipose tissue (WAT) in mice treated or not with sEV-Y. (**E**) Weight of BAT and WAT from old mice treated and untreated with sEV-Y. Data represents mean ± SEM of 5 old mice per condition.

Fafián-Labora *et al.* **Figure S6**

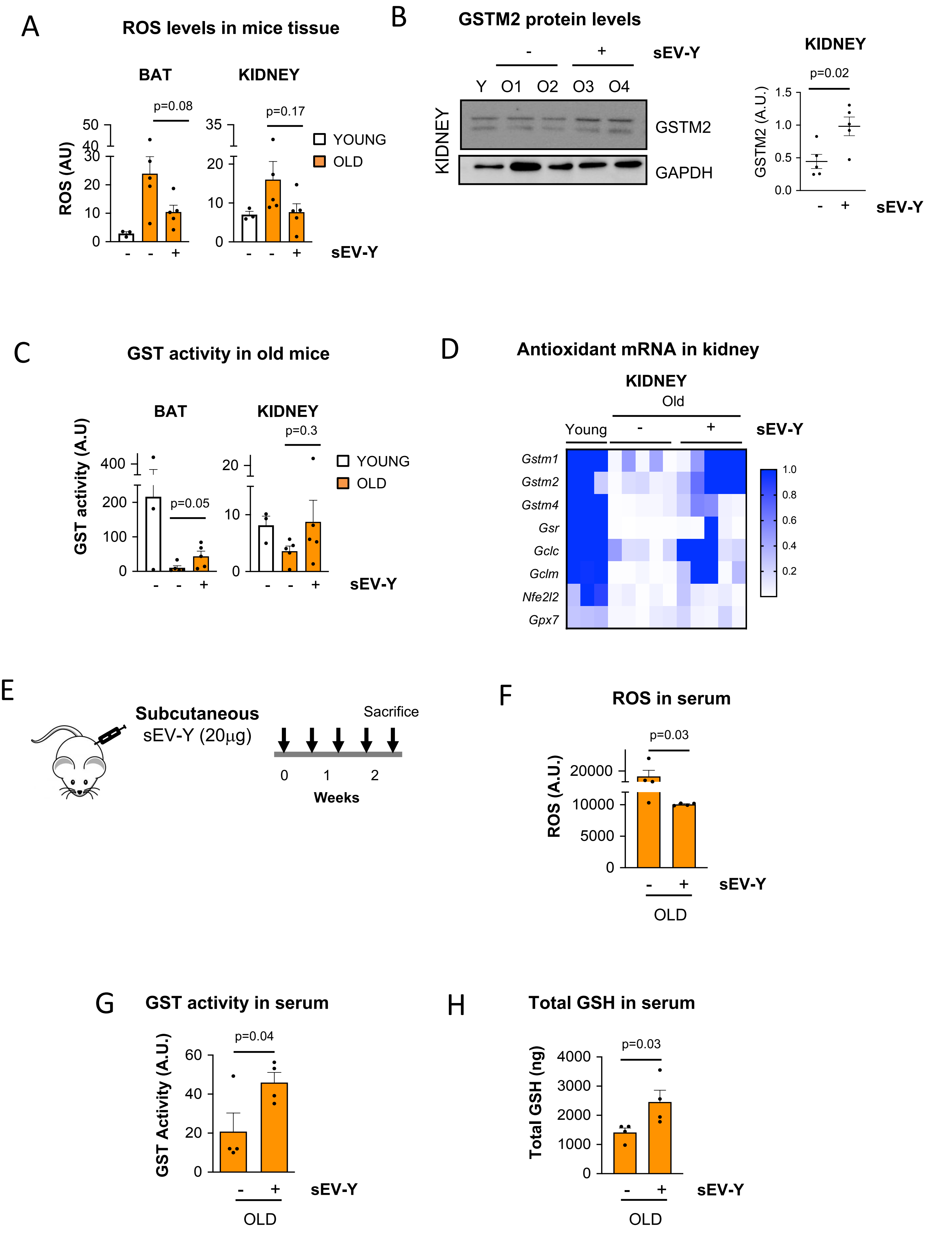

Figure S6. sEV-Y subcutaneous injection prevents ROS accumulation and induces an increase in the levels of GSH in old mice serum. Related to Figure 6.
(**A**) Quantification of ROS levels in BAT and kidney from young and old sEV-Y treated and untreated. Data represent the mean ± SEM of 3-5 mice. (**B**) Representative immunoblotting for GSTM2 in kidney. Densitometry for the mean ± SEM of GSTM2 in 5 old mice per condition is shown. (**C**) Relative GST activity in BAT and kidney from young and old mice treated or not with sEV-Y. Data represent the mean ± SEM of 3-5 mice and Mann-Whitney test was performed. (**D**) Heatmap representing the mean mRNA quantification of 3-5 mice determining different mRNA transcripts related to the antioxidant pathway in the kidney. (**E**) Diagram showing the experimental timeline where sEV-Y were injected subcutaneously for 2 weeks. (**F-H**) Analysis of (**F**) ROS, (**G**) GST activity and (**H**) GSH levels in serum from 4 old mice treated or not with sEV-Y. (**F, H**) Mann-Whitney analysis was performed.

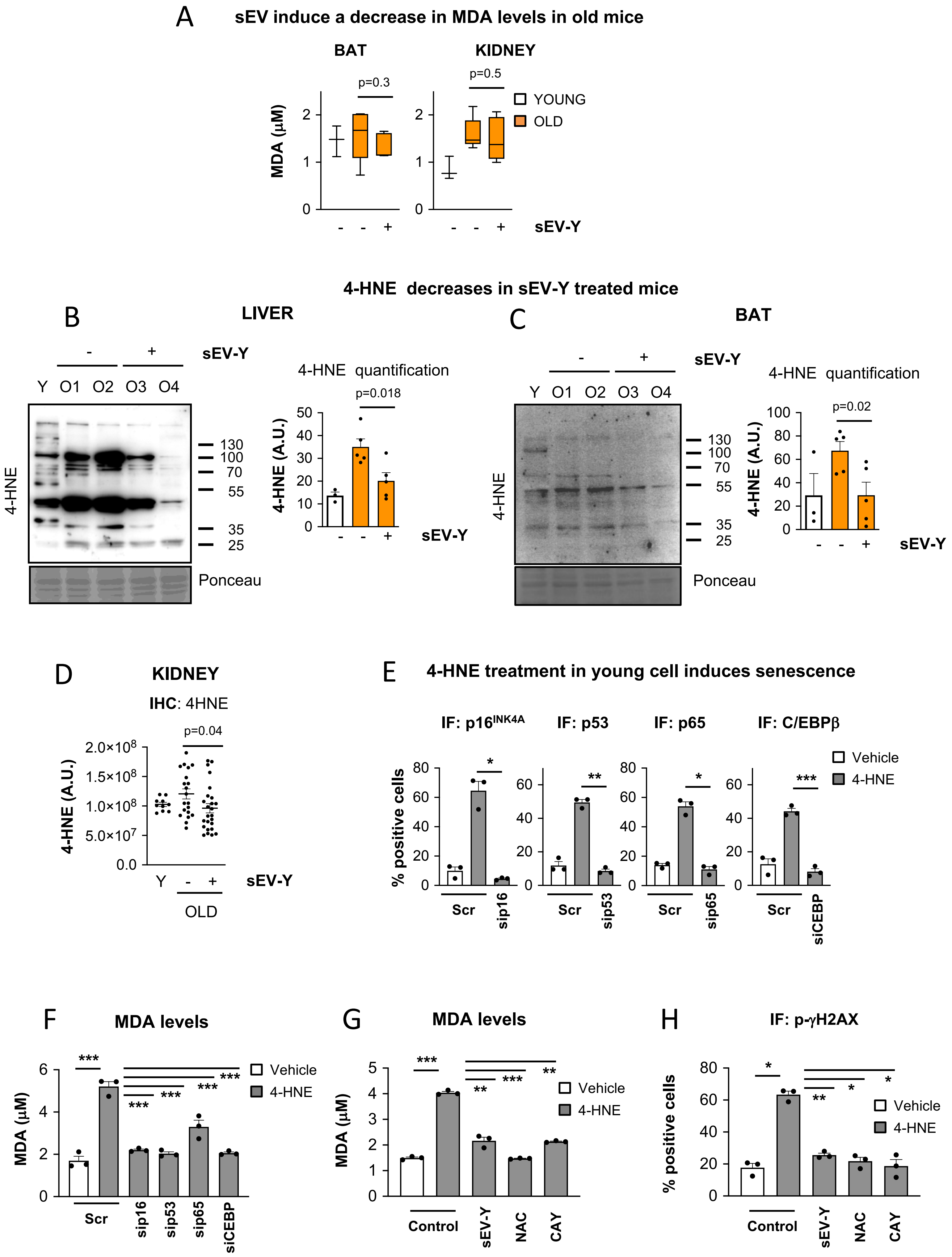
A
sEV induce a decrease in MDA levels in old mice
BAT
KIDNEY
p=0.3
p=0.5
YOUNG
OLD
MDA (µM)
sEV-Y
4-HNE decreases in sEV-Y treated mice
B
LIVER
C
BAT
sEV-Y
Y O1 O2 O3 O4
4-HNE
130
100
70
55
35
25
Ponceau
4-HNE quantification
p=0.018
p=0.02
4-HNE (A.U.)
D
KIDNEY
IHC: 4HNE
p=0.04
OLD
E
4-HNE treatment in young cell induces senescence
IF: p16INK4A
IF: p53
IF: p65
IF: C/EBPβ
% positive cells
Vehicle
4-HNE
Scr
sip16
sip53
sip65
siCEBP
F
MDA levels
G
MDA levels
H
IF: p-γH2AX
MDA (µM)
Control
sEV-Y
NAC
CAY

Figure S7. sEV-Y reduce the levels of oxidized lipids *in vivo* and *in vitro*. Related to Figure 7.

(**A**) MDA levels shown in BAT and kidney in young and old mice treated or not with sEV-Y. Data represent the mean ± SEM of 3 young and 5 old mice per condition (treated or not with sEV-Y). Mann-Whitney test was performed. (**B, C**) Representative immunoblotting for 4-HNE-adducted proteins and densitometry in (**B**) liver and (**C**) BAT tissue lysates from n=3 young and n=5 old mice treated or untreated with sEV-Y. Ponceau is shown as loading control. Graphs represent the mean ± SEM where Mann-Whitney test was performed. (**D**) Quantification of IHC staining for 4-HNE in kidney from young and old treated or not with sEV-Y from 3-5 mice. (**E**) IF to confirm the protein knockdown of the siRNA used. Vehicle and 4-HNE in $p16^{INK4A}$ graph are the same data as in **Figure 7F**. Data show the mean ± SEM of 3 independent experiments. (**F**) MDA levels in young HFFF2 cells treated with 4-HNE and different siRNA targeting various pathways that regulate senescence. Data show the mean ± SEM of 3 independent experiments. (**G**) MDA and (**H**) p-γH2AX levels with the different inhibitors: 20 µM CAY10576 targeting IKK pathway or 100nM NAC preventing DNA damage by inhibiting ROS. (**E-H**) Data represent the mean ± SEM of 3 independent experiments. One-Way ANOVA or student's t test was performed.

Table S1. MS dataset comparing sEV control *vs* senescent. Related to Figure 3.

Protein IDs	Protein names	Gene names	Fasta headers	id	Student's T-test DDIS_C_normal_fdr10%	Mean DDIS_log2(x/y)_Mean C	Student's T-test iRAS_C_normal_fdr10 %	Mean iRAS_log2(x/y)_Mean C
P28161	Glutathione S-transferase Mu 2	**GSTM2**	sp|P28161|GSTM2_HUMA	668	+	-4.54	+	-3.52
Q7RTV2	Glutathione S-transferase A5	**GSTA5**	sp|Q7RTV2|GSTA5_HUMA	1269	+	-4.50	+	-5.66
Q8IXL6	Extracellular serine/threonine protein kinase FAM20C	FAM20C	sp|Q8IXL6|DMP4_HUMAN	1295	+	-4.04	+	-3.20
P17516	Aldo-keto reductase family 1 member C4	AKR1C4	sp|P17516|AK1C4_HUMAI	553	+	-3.93	+	-4.56
P13929	Beta-enolase	ENO3	sp|P13929|ENOB_HUMAN	514	+	-3.49	+	-7.60
Q9BUD6	Spondin-2	SPON2	sp|Q9BUD6|SPON2_HUM/	1432	+	-3.48	+	-4.08
P48163	NADP-dependent malic enzyme	ME1	sp|P48163|MAOX_HUMAI	796	+	-3.43	+	-2.90
Q6ZNJ1	Neurobeachin-like protein 2	NBEAL2	sp|Q6ZNJ1|NBEL2_HUMAI	1252	+	-3.42	+	-4.31
Q9UBR2	Cathepsin Z	CTSZ	sp|Q9UBR2|CATZ_HUMAN	1528	+	-3.28	+	-4.22
P00746	Complement factor D	CFD	sp|P00746|CFAD_HUMAN	323	+	-3.23	+	-5.28
P50897	Palmitoyl-protein thioesterase 1	PPT1	sp|P50897|PPT1_HUMAN	834	+	-3.15	+	-2.80
Q14520	Hyaluronan-binding protein 2;Hyaluronan-binding protein 2 50 kDa heavy chain;Hyaluronan-binding protein :	HABP2	sp|Q14520|HABP2_HUMA	1133	+	-3.11	+	-4.09
P22352	Glutathione peroxidase 3	**GPX3**	sp|P22352|GPX3_HUMAN	613	+	-3.08	+	-4.09
P32754	4-hydroxyphenylpyruvate dioxygenase	HPD	sp|P32754|HPPD_HUMAN	717	+	-3.05	+	-3.65
P00451	Coagulation factor VIII;Factor VIIIa heavy chain, 200 kDa isoform;Factor VIIIa heavy chain, 92 kDa isoform;Fa	F8	sp|P00451|FA8_HUMAN (	312	+	-3.02	+	-3.12
P07738	Bisphosphoglycerate mutase	BPGM	sp|P07738|PMGE_HUMAI	411	+	-3.01	+	-2.80
O95834	Echinoderm microtubule-associated protein-like 2	EML2	sp|O95834|EMAL2_HUMA	304	+	-3.00	+	-3.90
P31146	Coronin-1A	CORO1A	sp|P31146|COR1A_HUMA	702	+	-2.99	+	-5.54
P00742	Coagulation factor X;Factor X light chain;Factor X heavy chain;Activated factor Xa heavy chain	F10	sp|P00742|FA10_HUMAN	322	+	-2.98	+	-5.73
P22105	Tenascin-X	TNXB	sp|P22105|TENX_HUMAN	610	+	-2.91	+	-4.20
Q14289	Protein-tyrosine kinase 2-beta	PTK2B	sp|Q14289|FAK2_HUMAN	1127	+	-2.89	+	-3.76
P55083	Microfibril-associated glycoprotein 4	MFAP4	sp|P55083|MFAP4_HUMA	878	+	-2.83	+	-3.38
P26572	Alpha-1,3-mannosyl-glycoprotein 2-beta-N-acetylglucosaminyltransferase	MGAT1	sp|P26572|MGAT1_HUM/	648	+	-2.78	+	-2.74
Q6EMK4	Vasorin	VASN	sp|Q6EMK4|VASN_HUMA	1234	+	-2.78	+	-5.09
P35590	Tyrosine-protein kinase receptor Tie-1	TIE1	sp|P35590|TIE1_HUMAN '	735	+	-2.74	+	-2.96
Q96C23	Aldose 1-epimerase	GALM	sp|Q96C23|GALM_HUMAI	1372	+	-2.71	+	-3.57
P08567	Pleckstrin	PLEK	sp|P08567|PLEK_HUMAN	436	+	-2.64	+	-6.03
O60462	Neuropilin-2	NRP2	sp|O60462|NRP2_HUMAN	221	+	-2.62	+	-3.46
Q92954	Proteoglycan 4;Proteoglycan 4 C-terminal part	PRG4	sp|Q92954|PRG4_HUMAN	1354	+	-2.62	+	-2.88
Q7LDG7	RAS guanyl-releasing protein 2	RASGRP2	sp|Q7LDG7|GRP2_HUMAN	1267	+	-2.59	+	-2.80
Q76LX8	A disintegrin and metalloproteinase with thrombospondin motifs 13	ADAMTS13	sp|Q76LX8|ATS13_HUMAI	1259	+	-2.54	+	-5.24
O43294	Transforming growth factor beta-1-induced transcript 1 protein	TGFB1I1	sp|O43294|TGFI1_HUMAI	202	+	-2.50	+	-2.58
P04424	Argininosuccinate lyase	ASL	sp|P04424|ARLY_HUMAN	360	+	-2.50	+	-2.62
P07954	Fumarate hydratase, mitochondrial	FH	sp|P07954|FUMH_HUMAI	421	+	-2.37	+	-2.35
P48506	Glutamate--cysteine ligase catalytic subunit	**GCLC**	sp|P48506|GSH1_HUMAN	799	+	-2.37	+	-2.93
Q16772	Glutathione S-transferase A3	**GSTA3**	sp|Q16772|GSTA3_HUMA	1201	+	-2.36	+	-5.25
P35580	Myosin-10	MYH10	sp|P35580|MYH10_HUMA	734	+	-2.33	+	-5.62
P12821	Angiotensin-converting enzyme;Angiotensin-converting enzyme, soluble form	ACE	sp|P12821|ACE_HUMAN /	496	+	-2.32	+	-4.97
Q16769	Glutaminyl-peptide cyclotransferase	QPCT	sp|Q16769|QPCT_HUMAN	1200	+	-2.31	+	-2.23
Q9UQ16	Dynamin-3	DNM3	sp|Q9UQ16|DYN3_HUMAI	1573	+	-2.25	+	-2.46
P04066	Tissue alpha-L-fucosidase	FUCA1	sp|P04066|FUCO_HUMAN	349	+	-2.25	+	-2.62
O60218	Aldo-keto reductase family 1 member B10	AKR1B10	sp|O60218|AK1BA_HUMA	218	+	-2.24	+	-3.51
P09455	Retinol-binding protein 1	RBP1	sp|P09455|RET1_HUMAN	448	+	-2.23	+	-2.70
Q06828	Fibromodulin	FMOD	sp|Q06828|FMOD_HUMA	1052	+	-2.23	+	-4.09
P00740	Coagulation factor IX;Coagulation factor IXa light chain;Coagulation factor IXa heavy chain	F9	sp|P00740|FA9_HUMAN (	321	+	-2.21	+	-3.85
P16885	1-phosphatidylinositol 4,5-bisphosphate phosphodiesterase gamma-2	PLCG2	sp|P16885|PLCG2_HUMAI	547	+	-2.21	+	-2.85
P14550	Alcohol dehydrogenase [NADP(+)]	AKR1A1	sp|P14550|AK1A1_HUMA	521	+	-2.20	+	-4.45
Q9H1B5	Xylosyltransferase 2	XYLT2	sp|Q9H1B5|XYLT2_HUMAI	1450	+	-2.18	+	-2.79
Q8IUX7	Adipocyte enhancer-binding protein 1	AEBP1	sp|Q8IUX7|AEBP1_HUMAI	1287	+	-2.14	+	-2.45
P03951	Coagulation factor XI;Coagulation factor XIa heavy chain;Coagulation factor XIa light chain	F11	sp|P03951|FA11_HUMAN	345	+	-2.13	+	-3.24
Q9H2A2	Aldehyde dehydrogenase family 8 member A1	ALDH8A1	sp|Q9H2A2|AL8A1_HUMA	1454	+	-2.13	+	-2.34
Q13418	Integrin-linked protein kinase	ILK	sp|Q13418|ILK_HUMAN Ir	1092	+	-2.12	+	-2.76
Q8NFP9;P50851	Neurobeachin;Lipopolysaccharide-responsive and beige-like anchor protein	NBEA;LRBA	sp|Q8NFP9|NBEA_HUMAI	1310	+	-2.10	+	-4.07
P00352	Retinal dehydrogenase 1	ALDH1A1	sp|P00352|AL1A1_HUMAI	310	+	-2.05	+	-3.89
Q9Y240	C-type lectin domain family 11 member A	CLEC11A	sp|Q9Y240|CLC11_HUMAI	1579	+	-2.02	+	-3.56

LEGEND

Proteins up FC > log-2 in Control(C) vs Senescent (both DDIS and iRAS)
Only statisticllly significant selected (+)
55 proteins selected

Dataset identifier **PXD010379**

Borghesan, M. et al. (2019) Small Extracellular Vesicles Are Key Regulators of Non-cell Autonomous Intercellular Communication in Senescence via the Interferon Protein IFITM3. Cell Rep. 27, 3956-3971.e6

Table S2. List of primers used in this study. Related to STAR Methods.

Target	Specie	Forward Primer	Reverse primer
RPS14	Human	CTGCGAGTGCTGTCAGAGG	TCACCGCCCTACACATCAAACT
CDKN2A	Human	CGGTCGGAGGCCGATCCAG	GCGCCGTGGAGCAGCAGCAGCT
CDKN1A	Human	CCTGTCACTGTCTTGTACCCT	GCGTTTGGAGTGGTAGAAATC
CDKN2B	Human	CGGTGTAAAATAGCAAGGGAAAT	TCAGTGCCTCTCATCTACTCTCC
IL8	Human	GAGTGGACCACACTGCGCCA	TCCACAACCCTCTGCACCCAGT
IL6	Human	CCAGGAGCCCAGCTATGAAC	CCCAGGGAGAAGGCAACTG
IL1A	Human	AGTGCTGCTGAAGGAGATGCCTGA	CCCCTGCCAAGCACACCCAGTA
IL1B	Human	TGCACGCTCCGGGACTCACA	CATGGAGAACACCACTTGT
MMP3	Human	AAGCTCTGAAAGTCTGGGAAGA	TCCCTGTTGTATCCTTTGTCCA
MMP9	Human	AACTTTGACAGCGACAAGAAGT	ATTCACGTCGTCCTTATGCAAG
CCL20	Human	GGCGAATCAGAAGCAGCAAGCAAC	ATTGGCCAGCTGCCGTGTGAA
Cdkn2a	Mouse	GTGTGCATGACGTGCGGG	GCAGTTCGAATCTGCACCGTAG
Cdkn1a	Mouse	TCCCGACTCTTGACATTGCT	TGCAGAAGGGGAAGTATGGG
Rps14	Mouse	GACCAAGACCCCTGGACCT	CCCCTTTTCTTCGAGTGCTA
Il1a	Mouse	CGCTTGAGTCGGCAAAGAAAT	TGGCAGAACTGTAGTCTTCGT
Il1b	Mouse	TGCCACCTTTTGACAGTGATG	TGATGTGCTGCTGCGAGATT
Il6	Mouse	TGATTGTATGAACAACGATGATGC	GGACTCTGGCTTTGTCTTTCTTGT
Cxcl1	Mouse	CTGGGATTCACCTCAAGAACATC	CAGGGTCAAGGCAAGCCTC
Gstm1	Mouse	AATTGGGATTGGTGCAGGGT	CGGACGTTCCAGTATCCCAG
Gstm2	Mouse	TTCTGTGATGGGCTGTGTCC	CACACAGGTTGTGCTTTCGG
Gstm4	Mouse	TAGCTCACGCTATTCGGCTG	CGGTCATAGTCAGGAGCGTC
Gclc	Mouse	ATGATAGAACACGGGAGGAGAG	TGATCCTAAAGCGATTGTTCTTC
Gclm	Mouse	TGACTCACAATGACCCGAAA	GATGCTTTCTTGAAGAGCTTCCT
Gsr	Mouse	AGTTCCTCACGAGAGCCAGA	CAGCTGAAAGAAGCCATCACT
Gpx7	Mouse	AGTTTGGCCAACAGGAACCA	CCGTCTGGGTCCACTAGGTA
Nfe2l2	Mouse	AAGAATAAAGTCGCCGCCCA	AGATACAAGGTGCTGAGCCG

Table S3. List of antibodies used in this study. Related to STAR Methods.

Antibody	Technique	Dilution
$p16^{INK4A}$	IF	1/500
β-Actin	WB	1/10000
$p21^{CIP}$	IF	1/500
p-γH2AX	IF	1/500
TSG101	WB	1/1000
ALIX	WB	1/1000
IL-8	IF	1/500
Calnexin	WB	1/1000
8-oxoG	IF	1/500
GSTM2	WB cells	1/1000
4-HNE	WB	1/1000
Myc tag	WB	1/1000
BrdU	IF	1/500
p65	IF	1/500
C/EBPβ	IF	1/500
